# *VPS35* mutation inhibits PINK1/parkin-mediated mitophagy via increased LRRK2 kinase activity

**DOI:** 10.1093/brain/awaf414

**Published:** 2025-10-30

**Authors:** Liselot Manders, Thibaut Heyninck, Dorien Imberechts, Bjørn Holst, Rejko Krüger, Wim Vandenberghe

**Affiliations:** Laboratory for Parkinson Research, Department of Neurosciences, KU Leuven, Leuven 3000, Belgium; Laboratory for Parkinson Research, Department of Neurosciences, KU Leuven, Leuven 3000, Belgium; Laboratory for Parkinson Research, Department of Neurosciences, KU Leuven, Leuven 3000, Belgium; Bioneer A/S, Hørsholm 2970, Denmark; Luxembourg Centre for Systems Biomedicine, University of Luxembourg, Esch-sur-Alzette L-4362, Luxembourg; Transversal Translational Medicine, Luxembourg Institute of Health, Strassen L-1445, Luxembourg; Parkinson Research Clinic, Centre Hospitalier de Luxembourg, Luxembourg L-1210, Luxembourg; Laboratory for Parkinson Research, Department of Neurosciences, KU Leuven, Leuven 3000, Belgium; Department of Neurology, University Hospitals Leuven, Leuven 3000, Belgium

**Keywords:** Parkinson’s disease, autophagy, mitochondrion, lysosome, induced pluripotent stem cell, RAB

## Abstract

The p.D620N mutation in *VPS35* causes an autosomal dominant form of Parkinson’s disease via mechanisms that are poorly understood. PINK1 and parkin, two proteins whose loss-of-function underlies autosomal recessive Parkinson’s disease, cooperate to mediate mitophagy, a quality control pathway for selective elimination of damaged mitochondria. PINK1/parkin-mediated mitophagy is disrupted by *LRRK2* mutations, which are the most prevalent cause of autosomal dominant Parkinson’s disease.

Here, we investigated whether the p.D620N *VPS35* mutation has an effect on PINK1/parkin-mediated mitophagy. We identified a novel family with autosomal dominant Parkinson’s disease caused by a p.D620N *VPS35* mutation. We cultured skin fibroblasts and induced pluripotent stem cell-derived dopaminergic neurons from the proband and from a second, unrelated Parkinson’s disease patient with the p.D620N *VPS35* mutation, and compared them with isogenic and non-isogenic control cells.

PINK1/parkin-mediated mitophagy was severely impaired in *VPS35* mutant fibroblasts and neurons, while non-selective, starvation-induced autophagy and lysosomal degradative capacity were preserved. siRNA-mediated *VPS35* knockdown rescued the mitophagy defect in *VPS35* mutant cells, whereas overexpression of wild-type VPS35 did not, suggesting a gain-of-function mechanism of the mutation. The *VPS35* mutation did not interfere with activation of PINK1 or parkin after mitochondrial depolarization, but impaired mitochondrial recruitment of the autophagy receptor optineurin. LRRK2 kinase activity was increased in the *VPS35* mutant cells, as shown by enhanced levels of the T73-phosphorylated form of the LRRK2 substrate RAB10. The enhanced level of phosphorylated RAB10 in *VPS35* mutant cells was decreased by treatment with LRRK2 kinase inhibitors and by *VPS35* knockdown. Importantly, the mitophagy defect of *VPS35* mutant fibroblasts and neurons was fully rescued by LRRK2 kinase inhibitors as well as by overexpression of PPM1H, a phosphatase that dephosphorylates multiple RAB substrates of LRRK2. Finally, *in situ* proximity ligation experiments revealed that endogenous VPS35 and LRRK2 are proximity partners in human dopaminergic neurons and that this proximity relationship is enhanced by the *VPS35* mutation.

In conclusion, the *VPS35* mutation impairs PINK1/parkin-mediated mitophagy via a gain-of-function mechanism that involves stimulation of LRRK2 kinase activity. Thus, a VPS35/LRRK2 axis linked to dominant Parkinson’s disease intersects with a pathway mediated by proteins encoded by the recessive Parkinson’s disease genes.

## Teaser

*VPS35* and *LRRK2* mutations cause autosomal dominant Parkinson’s disease via mechanisms that are poorly understood. Manders *et al.* show in patient fibroblasts and neurons that a VPS35/LRRK2 axis intersects with a mitochondrial quality control pathway mediated by proteins linked to autosomal recessive Parkinson’s disease.

## Introduction

Parkinson’s disease is a highly prevalent neurodegenerative condition characterized clinically by a wide spectrum of progressive motor and non-motor symptoms.^1^ In most patients with Parkinson’s disease, the aetiology is poorly understood and likely multifactorial, but in approximately 5% of cases, a monogenic cause can be identified.^2^ Mutations in *PRKN*, *PINK1* and *PARK7* cause autosomal recessive Parkinson’s disease, while mutations in *LRRK2*, *SNCA*, *VPS35*, *CHCHD2* and *RAB32* cause autosomal dominant forms.^3-5^ Moreover, heterozygous variants in *GBA1* are a common genetic risk factor for Parkinson’s disease.^2,3^

Parkinson’s disease-linked loss-of-function mutations in *PRKN*, *PINK1* and *PARK7* all exert an inhibitory effect on a quality control pathway for selective autophagic elimination of damaged mitochondria (mitophagy). The E3 ubiquitin ligase parkin (encoded by *PRKN*) and the mitochondrial kinase PINK1 closely collaborate to generate poly-ubiquitin chains on damaged mitochondria.^6,7^ Mitochondrial damage and depolarization are sensed by PINK1 and result in PINK1 accumulation on the outer mitochondrial membrane (OMM). PINK1 then phosphorylates ubiquitin and the ubiquitin-like domain of parkin, which is necessary for mitochondrial translocation of parkin and for parkin activation.^6,7^ Activated parkin ubiquitinates a large number of OMM proteins, initiating a positive feedback loop in which PINK1 phosphorylates additional ubiquitin and parkin molecules, leading to even more parkin activity on the OMM. Ubiquitin-coated mitochondria are recognized by autophagy receptors, which act as bridges between ubiquitinated mitochondrial cargo and the autophagy machinery, eventually allowing selective degradation of mitochondrial material inside lysosomes.^6,7^ DJ-1, encoded by *PARK7*, is essential for PINK1/parkin-mediated mitophagy, more specifically for optimal mitochondrial recruitment of the autophagy receptor optineurin, downstream of PINK1 and parkin activation.^8^ Importantly, *LRRK2* mutations, the most prevalent cause of autosomal dominant Parkinson’s disease and of monogenic Parkinson’s disease overall, also interfere with mitophagy.^9-18^ Parkinson’s disease-linked *LRRK2* mutations increase LRRK2 kinase activity in cells,^19^ and LRRK2 kinase inhibition rescued the mutant LRRK2-induced mitophagy defect in some,^10,11,13,14,16^ but not all,^9,15,17,18^ studies.

*VPS35* is another autosomal dominant Parkinson’s disease gene.^20,21^ Of the reported variants in *VPS35*, only one (p.D620N) is considered definitely pathogenic.^22^ Although the D620N mutation is very rare and detailed clinical descriptions are scarce, available data suggest that *VPS35*-linked Parkinson’s disease is clinically similar to idiopathic disease, apart from a slightly younger median age at onset.^22^ VPS35 is a component of the retromer, a protein complex responsible for retrieval of transmembrane proteins from endosomes to the plasma membrane or trans-Golgi network.^23^ How the D620N mutation causes neurodegeneration is not clear yet. Whether this mutation causes Parkinson’s disease through loss of the retromer function of VPS35 (via haplo-insufficiency or a dominant negative mechanism) or through a toxic gain-of-function is still debated.^23^ Interestingly, the D620N mutation induces a strong increase of LRRK2 kinase activity in mouse embryonic fibroblasts as well as in neutrophils and monocytes from Parkinson's disease patients, suggesting that this mutation acts upstream of LRRK2.^24^ Given that increased LRRK2 kinase activity in *LRRK2* mutant cells inhibits mitophagy,^10,11,13,14,16^ this raises the possibility that the *VPS35* mutation could also affect mitophagy.

Therefore, we designed a study to determine the effect of the endogenous p.D620N *VPS35* mutation on PINK1/parkin-dependent mitophagy in human fibroblasts and neurons.

## Materials and methods

### Antibodies

The following primary antibodies were used for western blot (WB), immunofluorescence (IF), proximity ligation assays (PLA) and flow cytometry (FC): mouse anti-β-actin (WB, 1:5000; Sigma, A5441), rabbit anti-HSP60 (WB, 1:1000; IF, 1:1000; Abcam, ab53109), mouse anti-ATP5F1B (IF, 1:200; Abcam, ab14730), mouse anti-LC3B (WB, 1:2000; Novus Biologicals, NB100-2220), rabbit anti-PINK1 (WB, 1:1000; Novus Biologicals, BC100-494), mouse anti-mitofusin 2 (MFN2) (WB, 1:1000; Abcam, ab56889), mouse anti-ubiquitin (WB, 1:50; Santa Cruz, sc-8017), rabbit anti-phospho-ubiquitin^S65^ (WB, 1:1000; Sigma, ABS1513-I), rabbit anti-optineurin (IF, 1:250; Invitrogen, 711879), rabbit anti-RAB10 (WB, 1:1000; Cell Signaling Technology, 8127S), rabbit anti-phospho-RAB10^T73^ (WB, 1:500; Abcam, ab230261), rabbit anti-tyrosine hydroxylase (TH) (IF, 1:500; Sigma, AB152), sheep anti-TH (IF, 1:300; Thermo Fisher, PA1-4679), mouse anti-MAP2 (IF, 1:500; Sigma, M1406), rabbit anti-mGFP (WB, 1:5000; Origene, TA150122), rabbit anti-TurboGFP (tGFP) (WB, 1:2000; Origene, TA150071), rabbit anti-VPS35 (WB, 1:1000; Abcam, ab157220), mouse anti-VPS35 (PLA; 1:100; Abcam, ab57632), rabbit anti-LRRK2 (PLA, 1:200; IF, 1:200; Abcam, ab133518) rabbit anti-LRRK2 (PLA, 1:200; Abcam, ab133476), mouse anti-LAMP1 (IF, 1:100; BD Biosciences, 555798), rabbit anti-PRDX1 (PLA, 1:250; Sigma, HPA007730), rabbit anti-calnexin (PLA, 1:500; Enzo, ADI-SPA-860), mouse anti-GM130 (PLA, 1:200 ; BD Biosciences, 610823), rabbit anti-Oct4 (IF, 1:100; Santa Cruz, sc-9081), goat anti-Oct4 (FC, 1:100; Abcam, ab27985), rabbit anti-Nanog (IF, 1:300; Thermo Fisher, PA1-097X), mouse anti-Tra-1-60 (IF, 1:200; Cell Signaling Technology, 4746), mouse anti-Tra-1-81 (IF, 1:200; Cell Signaling Technology, 4745), mouse anti-Tra-1-81 (FC, 1:200; Biolegend, 330702), rabbit anti-Sox2 (IF, 1:500; Merck Millipore, AB5603), rabbit anti-Sox2 (FC, 1:100; Abcam, ab97959), mouse anti-SSEA4 (IF, 1:200; Santa Cruz, sc-21704), mouse anti-SSEA4 (FC, 1:50; Biolegend, 330402), mouse anti-SSEA1 (FC, 1:25; BD Pharmingen, 560142), mouse anti-Sox1 (FC, 1:25; BD Pharmingen, 561592), mouse anti-PAX6 (FC, 1:50; BD Pharmingen, 562249), mouse anti-CD34 (FC, 1:25; BD Pharmingen, 555822), mouse anti-CD56 (FC, 1:25; BD Pharmingen, 555518), mouse anti-CD184 (FC, 1:25; BD Pharmingen, 555974) and mouse anti-Sox17 (FC, 1:50; BD Pharmingen, 562594). Peroxidase-linked secondary antibodies for WB were from Sigma (SAB3700934, SAB3701095). Secondary antibodies for IF were donkey anti-mouse Alexa Fluor-555 (Thermo Fisher, a31570), donkey anti-rabbit Alexa Fluor-488 (Thermo Fisher, a21206), donkey anti-sheep Alexa Fluor-488 (Thermo Fisher, a11015), goat anti-rabbit Alexa Fluor-488 (Thermo Fisher, a11034), goat anti-mouse (IgM) Alexa Fluor-555 (Thermo Fisher, a21426) and goat anti-mouse (IgG) Alexa Fluor-555 (Thermo Fisher, a21424). Secondary antibodies for FC were donkey anti-mouse Alexa Fluor-488 and Alexa Fluor-647 (Thermo Fisher, a21202, a31571), donkey anti-rat Alexa Fluor-594 (Invitrogen, a21209), donkey anti-goat Alexa Fluor-488 (Invitrogen, a11055) and donkey anti-rabbit Alexa Fluor-488 (Thermo Fisher, a21206).

### cDNAs, siRNAs and lentivirus

pCMV6-AC-GFP vectors containing cDNA for tGFP-tagged VPS35 and tGFP-tagged PPM1H were purchased from Origene (RG210597 and RG217548, respectively). The mito-Keima construct (mt/mKeima/pIND[SP1]) was a gift from Dr. A. Miyawaki (RIKEN Brain Science Institute, Japan) and the Keima construct (mKeima-Red-N1) was a gift from Dr. M. Davidson (Addgene, 54597). Optineurin-EGFP was a gift from Dr. B. Yue (Addgene, 27052). pEGFP-C1 vector was a gift from Dr P. Vangheluwe (KU Leuven). *VPS35* siRNA1 was a pool of 4 siRNAs with the following target sequences: 5′-GAACAUAUUGCUACCAGUA-3′, 5′-GAAAGAGCAUGAGUUGUUA-3′, 5′-GUUGUAAACUGUAGGGAUG-3′ and 5′-GAACAAAUUUGGUGCGCCU-3′ (Horizon Discovery, L-010894-00-0005). *VPS35* siRNA2 (Qiagen, SI04279296) had the target sequence 5′-AGCACTTATCTTGGCTACTAA-3′. The target sequence of control siRNA (Qiagen, 1027417) was 5′-AATTCTCCGAACGTGTCACGT-3′. pLenti-C-mGFP lentiviral particles encoding human PPM1H, pLenti-C-mGFP-P2A-Puro lentiviral particles encoding human VPS35 and pLenti-C-mGFP-P2A-Puro lentiviral control particles were purchased from Origene (RC217548L2V, RC210597L4V and PS100093V, respectively). Cloning of Keima and mito-Keima cDNA into lentivirus and production of lentiviral particles were described before.^8^

### Skin-derived fibroblasts from Parkinson’s disease patients and healthy controls

After written informed consent, we performed skin biopsy from the medial upper limb to obtain fibroblasts from a 62-year-old male Parkinson’s disease patient with heterozygous p.D620N *VPS35* mutation (*VPS35* patient 1) and from four healthy controls: a 68-year-old male (Ctrl1), a 57-year-old female (Ctrl2), a 74-year-old male (Ctrl3) and a second 68-year-old male (Ctrl4). All healthy controls had a normal clinical neurological exam. We also used previously collected skin fibroblasts from a second, unrelated, 76-year-old male Parkinson’s disease patient with the heterozygous p.D620N *VPS35* mutation (*VPS35* patient 2),^25^ a 46-year-old female Parkinson’s disease patient with compound heterozygous *PRKN* mutations (deletion of exon 2 and duplication of exon 6)^8^ and a 67-year-old male Parkinson’s disease patient with a heterozygous p.R1441C *LRRK2* mutation.^13^  *VPS35* patients 1 and 2, the patient with *PRKN* mutations and the patient with the p.R1441C *LRRK2* mutation did not have other pathogenic variants in known Parkinson’s disease genes. In *VPS35* patient 1, the *PRKN* patient and the *LRRK2* patient, samples for next-generation sequencing analysis were prepared using the HaloPlex HS panel (custom Parkinson panel v1) (Agilent). All exons and flanking intronic sites of *PINK1*, *PRKN*, *PARK7*, *SNCA*, *LRRK2*, *VPS35* and *GBA1* were sequenced on a MiSeq platform (Illumina). Variants were annotated using RefSeq v88, genome build GRCh38 and Alissa Interpret v5.3 (Agilent). In addition, multiplex ligation dependent probe amplification (SALSA MLPA P051-D1, MRC Holland) was performed to detect genomic deletions and duplications in *PINK1*, *PRKN*, *PARK7* and *SNCA*. The methods of genetic analysis of *VPS35* patient 2 (previously reported as patient #2610) were described before.^26 18^F-FE-PE2I PET imaging of *VPS35* patient 1 was performed as previously described.^27^ All procedures were approved by the local ethics committee and were in accordance with the latest version of the World Medical Association Declaration of Helsinki. Fibroblasts were cultured as previously described.^8,13^ Cultures were repeatedly tested for *Mycoplasma* by PCR, and tests were always negative. Fibroblast experiments were performed at passage numbers 4–14. Within the same experiment control and mutant fibroblasts were used at similar passage numbers.

### Induced pluripotent stem cell generation, quality control and differentiation to dopaminergic neurons

Fibroblasts from *VPS35* patient 1 were reprogrammed to induced pluripotent stem cells (iPSCs) at Bioneer (Hørsholm, Denmark) by electroporation (Lonza Neon Transfection System) of the reprogramming factors Oct4, Sox2, Lin28, Klf4 and L-Myc using the Epi5 Episomal iPSC Reprogramming kit (Thermo Fisher, A15960) according to the vendor’s protocol. At 21–28 days after reprogramming, appearing colonies were picked manually and replated. After five further passages, three clones growing as typical pluripotent colonies with no or very little visible differentiation were chosen for DNA isolation and qRT-PCR to test for absence of the reprogramming plasmids. As a negative control, DNA from the original fibroblasts was included in the PCR reactions. One clone (Clone 1) with demonstrated absence of reprogramming plasmids was confirmed to have normal karyology by G-band analysis (banding quality of 350) (Supplementary Fig. 1A), and a cell bank of Clone 1 was generated. One cryopreserved vial of Clone 1 was thawed, and the following quality control analyses were performed at Bioneer. To test for sterility, 100 μl of supernatant was transferred to 10 ml of LB medium without antibiotics. After 48 h of incubation in a shaker at 37°C, the LB medium was visually still clean, indicating that no bacterial contamination was present. *Mycoplasma* testing by PCR was negative. On Days 1 and 2 after thawing, typical iPSC morphology was observed by light microscopy (Supplementary Fig. 1B). G-banding at passage 9 after thawing again showed a normal karyotype. Single nucleotide polymorphism (SNP) analysis at passage 9 using iCS-digital PSC (Stem Genomics) did not detect any recurrent genomic abnormalities. Short tandem repeat (STR) analysis on extracted DNA using the AmpFLSTR Identifiler PCR Amplification kit (Applied Biosystems) confirmed genomic identity of the generated iPSC line with the original fibroblasts (Supplementary Fig. 1C). Sanger sequencing of the relevant region of the *VPS35* gene confirmed that both the iPSC line and the parental fibroblasts had a heterozygous G>A mutation leading to a D620N change. Pluripotency marker analysis was performed using flow cytometry. Cells were stained for flow cytometry using a Foxp3/Transcription Factor Staining Buffer Set (eBioscience, 00-5523-00) combined with an Intracellular Fixation and Permeabilization Buffer Set (eBioscience, 88-8824-00) according to the manufacturer’s instructions. Cells were stained for nuclear (Sox2, Oct4) and surface (SSEA4, Tra-1-81) pluripotency markers and for the differentiation surface marker SSEA1 as a control for possible differentiation. Cells without antibody labelling served as a technical negative control. Cells were analysed on a BD Accuri C6 Flow Cytometer (BD Biosciences) using FlowJo software (BD Biosciences). The line showed high expression of Sox2, Oct4, SSEA4 and Tra-1-81, and approximately 5% of cells were stained slightly positive for SSEA1 (Supplementary Fig. 1D). Previously described culture conditions were used to drive ectodermal, endodermal and mesodermal differentiation,^28^ followed by flow cytometry to confirm trilineage differentiation potential via detection of ectodermal (PAX6, Sox1), endodermal (CD184, Sox17) and mesodermal (CD34, CD56) markers (Supplementary Fig. 1E–G).

CRISPR-Cas9 technology was used at Bioneer to generate a D620N gene-corrected, isogenic control iPSC line for Clone 1 of *VPS35* patient 1. Clone 1 cells were nucleofected (Lonza Neon Transfection System) with CRISPR complexes formed by Cas9 protein (Alt-R S.p. HiFi Cas9 Nuclease V3; IDT, 1081061) and single guide RNA (sgRNA), and a single-stranded oligodeoxynucleotide (ssODN) as homologous template. The sequence of the sgRNA was: 5′-GATGGCAGCTAGCTGTGCTTTGG-3′. The sequence of the ssODN was: 5′-TCCTTTCTGCTTTATGTTCACTAGGCATTTTCTCTGTATGAAGATGAAATCAGCGATTCGAAGGCACAGCTAGCTGCCATCACCTTGATCATTGGCACTTTTGAAAGGATGAAGTGCTTC-3′. PCR to investigate potentially gene-edited clones was performed using the following primers: 5′-AGAGGATGGTTGGTCCTTGA-3′ and 5′-TCCTCCCCATTTTTGTCCGT-3′. The PCR product was investigated with a restriction fragment length polymorphism (RFLP) assay using the enzyme *Bst*BI (Supplementary Fig. 2A). Clones with the desired RFLP pattern were analysed with Sanger sequencing to confirm the correct base change. One homozygous gene-corrected clone was identified (IsoCtrl Clone 1), expanded and banked. After banking, one vial of IsoCtrl Clone 1 was thawed and successfully underwent quality control analysis at Bioneer (sterility and *Mycoplasma* testing, morphological evaluation, G-banding, SNP analysis, STR analysis, Sanger sequencing to confirm the desired base change, pluripotency analysis), as described above (Supplementary Fig. 2B–E).

Generation and quality control analysis of the iPSC line from *VPS35* patient 2 (LSCBi001-A) were described previously.^25^

Fibroblasts from Ctrl4 were reprogrammed to iPSCs at KU Leuven via Sendai virus-mediated overexpression of Yamanaka transcription factors Oct4, Sox2, Klf4 and C-myc. Three iPSC clones were generated and checked for clearance of the Sendai virus. All Ctrl4 iPSC clones successfully underwent quality controls at KU Leuven using methods that were previously described in detail^8,29^: immunofluorescent staining for pluripotency markers (Supplementary Fig. 3A), analysis of expression of self-renewal genes and trilineage differentiation potential (Supplementary Fig. 3B), SNP analysis to confirm genomic identity with the initial fibroblasts (Supplementary Fig. 3C), and array comparative genomic hybridization that showed no significant genome-wide aberrations.

iPSCs were differentiated to dopaminergic neurons using a protocol that was previously described in detail,^8,29^ based on methods developed by Kriks *et al*.^30^

### Transfection and lentiviral transduction

Fibroblasts were transiently transfected with 20 nM siRNA or 0.3 µg/µl cDNA (final concentrations) using the Neon Transfection System (Invitrogen, MPK1096) or Neon NxT Electroporation System (Invitrogen, NEON1S) according to the manufacturer’s instructions. Neurons were transduced with lentivirus on Days 48–50 after neuronal induction.

### Mito-Keima and Keima imaging

Live ratiometric mito-Keima and Keima imaging was performed as previously described in fibroblasts^8,13,29^ and iPSC-derived neurons.^8,29^ Fibroblasts were transiently transfected with mito-Keima or Keima cDNA. Neurons were transduced on Day 48 after neuronal induction with lentivirus containing mito-Keima or Keima. Cells were treated with valinomycin (Sigma, V3639), oligomycin (Sigma, 495455), antimycin A (Sigma, A8674), bafilomycin A1 (Abcam, ab120497), PF-06447475 or MLi-2 (Tocris, 5716, 5756). Starvation-induced autophagy was triggered by incubation in Earle’s balanced salt solution (EBSS) (Sigma, E2888). Cells were analysed using Leica TCS SP5 II or SP8 confocal microscopes, both with a 63× objective lens (HCPL APO 63x/1.4 CS2). Keima and mito-Keima were imaged in two channels via two sequential excitations (458 nm, green; 543 nm, red) and using a 600 to 695 nm emission range. Images from random microscopic fields were captured and analysed by an investigator blinded to genotype and experimental condition. Ratio (543/458) images were created using the Ratio Plus plugin in ImageJ. High (543/458) mito-Keima or Keima ratio areas were segmented and quantified with the Analyze Particles plugin in ImageJ. The parameter high mito-Keima (543/458) ratio area/total mitochondrial area was used as an index of mitophagy. Total mitochondrial area was determined by segmenting the area of total emission at 458 nm excitation in the original image, which was also quantified using the Analyze Particles plugin. The parameter high Keima (543/458) ratio area/total cell area was used as an index of autophagy. Total cell area was quantified by measuring the total Keima emission area at 458 nm excitation using the Analyze Particles plugin. For easy visualization of the level of mitophagy or autophagy in figures, a red hot lookup table (LUT) was applied to the ratio images in ImageJ. In some experiments, mito-Keima was imaged in cells that also expressed EGFP, tGFP or mGFP, as previous work showed that this can be done without significant cross-excitation, cross-detection or resonance transfer.^31^

### Western blot and immunocytochemistry

For western blot, cells were washed with ice-cold PBS, removed with a scraper and resuspended in PBS (350 mM NaCl, 2.7 mM KCl, 10.2 mM Na_2_PO_4_, 1.75 mM KH_2_PO_4_, pH 7.4) with 1% Triton X-100 (Sigma, 93443), 1% phosphatase inhibitor (Thermo Fisher, 78420) and 1% protease inhibitor (Thermo Fisher, 78430). After solubilization on ice for 30 min, insoluble material was removed by centrifugation at 20 000*g* for 5 min. Protein concentrations were determined using Bio-Rad Protein assay, followed by western blot as previously described.^8,13^

For immunocytochemistry, cells grown on poly-D-lysine (Sigma, P6407)-coated coverslips were washed with PBS, fixed in 4% paraformaldehyde in PBS for 30 min, washed with PBS and blocked and permeabilized for 45 min in PBS containing 5% horse serum and 0.1% Triton X-100. Cells were incubated for 1 h with primary antibodies in PBS containing 0.1% Triton X-100 and 2% horse serum. After washing, secondary antibodies were applied for 1 h in PBS containing 0.1% Triton X-100 and 2% horse serum. Nuclei were stained with TOTO-3 iodide (1 µM in the secondary antibody solution; Thermo Fisher, T3604). Cells were mounted onto microscope slides with Vectashield (Vector Laboratories, H-1000-10) or Prolong Glass Antifade Mountant (Thermo Fisher, P36982). Confocal images with 0.5 µm slice thickness were acquired at room temperature with a Leica TCS SP5 II or SP8 confocal microscope described earlier. Random images were captured and analysed by an investigator blinded to genotype and experimental condition.

### Proximity ligation assay

Cells were fixed and permeabilized as described for immunostaining. Antibody incubation and probe amplification were performed according to the manufacturer’s instructions (Duolink In Situ Red Starter Kit Mouse/Rabbit, Sigma, DUO 92101). To combine the proximity ligation assay (PLA) with immunostaining of dopaminergic neurons, cells were incubated simultaneously with sheep anti-TH and primary antibodies used for PLA. Following the washing steps, cells were incubated with secondary antibody for immunofluorescence together with the PLA probes. Samples were covered with the Duolink detection solution, washed and mounted according to the PLA protocol. Confocal images with 0.5 µm slice thickness were acquired at room temperature with a Leica TCS SP8 confocal microscope (described earlier) and analysed by an investigator blinded to genotype and experimental condition. To quantify the area of PLA signal, the colocalization image creator plugin in ImageJ was used.^29,32^ Z-stack images were segmented by applying an intensity threshold and a binary image was generated in which the segmented z-layers were projected. Subsequently, the area of the PLA signal in the binary z-projection image was measured using the Analyze Particles plugin in ImageJ and divided by total TH-positive area.^29,32^

### DQ-BSA assay

DQ-Red BSA (10 µg/ml) (Invitrogen, D12051) was applied to cells 2 h prior to live imaging. LysoTracker Green (100 nM) (Invitrogen, L7526) was added just before live imaging. Confocal images with 0.5 µm slice thickness were acquired at 37°C and 5% CO_2_, using a Nikon TiE A1R confocal microscope equipped with a 60× objective lens (DIC APO 60×/1.40 N2) and 405, 488, 543 and 633 nm diode lasers, and analysed by an investigator blinded to genotype and experimental condition. To quantify the area of DQ-Red BSA signal, the colocalization image creator plugin in ImageJ was used as described above for determination of PLA signal area.

### Statistics

Significance of differences was analysed with two-tailed Student’s *t*-test for comparison between two groups and with one-way ANOVA and *post hoc* Tukey’s test for comparison between more than two groups (GraphPad Prism 9, GraphPad Software, Inc.). Data were presented as mean ± standard error of the mean. A *P*-value of <0.05 was considered statistically significant. *n* indicates the number of independent biological replicates. In experiments on iPSC-derived neurons, each of the biological replicates was performed on cells from a different differentiation batch. In imaging experiments, the number of cells analysed in each biological replicate is indicated in the figure legend. In the graphs, each dot represents one biological replicate.

## Results

### A family with autosomal dominant Parkinson’s disease caused by *VPS35* mutation

**Figure 1:**
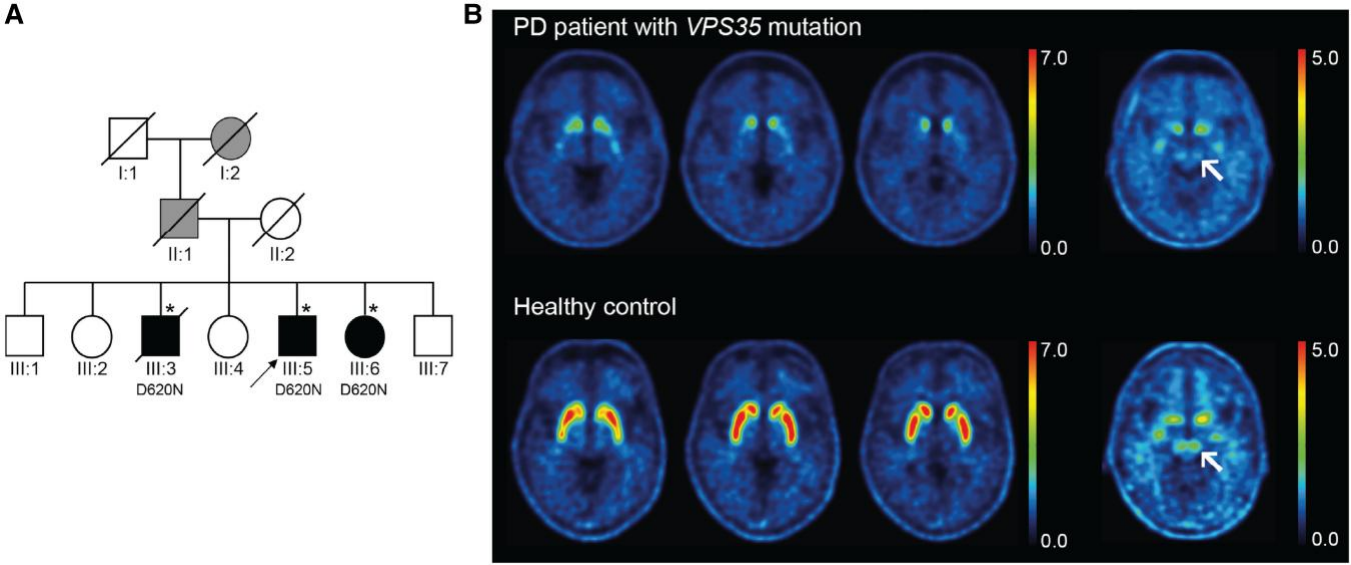
**Pedigree and dopamine transporter imaging of *VPS35* patient 1.** (**A**) Pedigree. Circles and squares denote females and males, respectively. Black filled symbols indicate individuals with a diagnosis of Parkinson’s disease based on clinical examination by the principal investigator. Grey filled symbols indicate individuals with Parkinson’s disease diagnosis based on report by the proband. Diagonal bars through symbols denote deceased individuals. Asterisks denote genotyped individuals. The arrow indicates the proband (*VPS35* patient 1). (**B**) Transverse ^18^F-FE-PE2I standardized uptake value ratio (SUVR) images of *VPS35* patient 1 (III:5) at age 60 years and a male 61-year-old healthy control. The healthy control was scanned in the context of a previously published study.^27^ Occipital cortex was used as reference region for standardized uptake value ratio (SUVR) calculation. Colour bar shows SUVR values. The white arrow points to uptake in the substantia nigra.

We identified a novel Flemish family with autosomal dominant Parkinson’s disease caused by a p.D620N *VPS35* mutation (Fig. 1A). The index patient (*VPS35* patient 1, III:5) was a 61-year-old right-handed male, who developed gradual impairment of left hand and leg mobility at the age of 55 years. He reported no tremor or dystonia. He had no prominent non-motor problems except for recurrent depressive episodes. Pramipexole (1.05 mg daily) was initiated 2 years after symptom onset but mainly induced excessive daytime somnolence. At the age of 61, pramipexole was replaced by levodopa (100 mg t.i.d.), with a favourable effect on his motor symptoms and somnolence. Clinical exam at the age of 61 showed mild hypomimia and aprosodia and subtle postural tremor of the upper limbs, more so on the left, without rest tremor. There was mild cogwheel rigidity of the left arm and mild bradykinesia of the left hand and foot. Arm swing was reduced on the left, but gait was otherwise normal. There was no freezing of gait. Postural reflexes were normal. The Movement Disorder Society-Unified Parkinson’s Disease Rating Scale (MDS-UPDRS) III score (off medication) was 16. An overview of the patient’s clinical scores on motor and non-motor scales is shown in Supplementary Table 1. Structural brain MRI was normal. PET with dopamine transporter ligand ^18^F-FE-PE2I showed a severe, symmetrical reduction of uptake in the striatum, especially in the putamen, as well as in the substantia nigra (Fig. 1B). At the age of 62, he developed mild wearing off and peak-dose dyskinesias under treatment with 350 mg levodopa and 1 mg rasagiline daily. Genetic analysis revealed a heterozygous c.1858G>A (p.D620N) mutation in *VPS35*. Skin biopsy was performed at age 62. Two of the patient’s five siblings also had Parkinson’s disease (III:3 and III:6, with motor symptom onset at 51 and 52 years, respectively) with a heterozygous c.1858G>A (p.D620N) *VPS35* mutation (Fig. 1A).

### *VPS35* mutation disrupts PINK1/parkin-mediated mitophagy in human fibroblasts and iPSC-derived neurons

**Figure 2:**
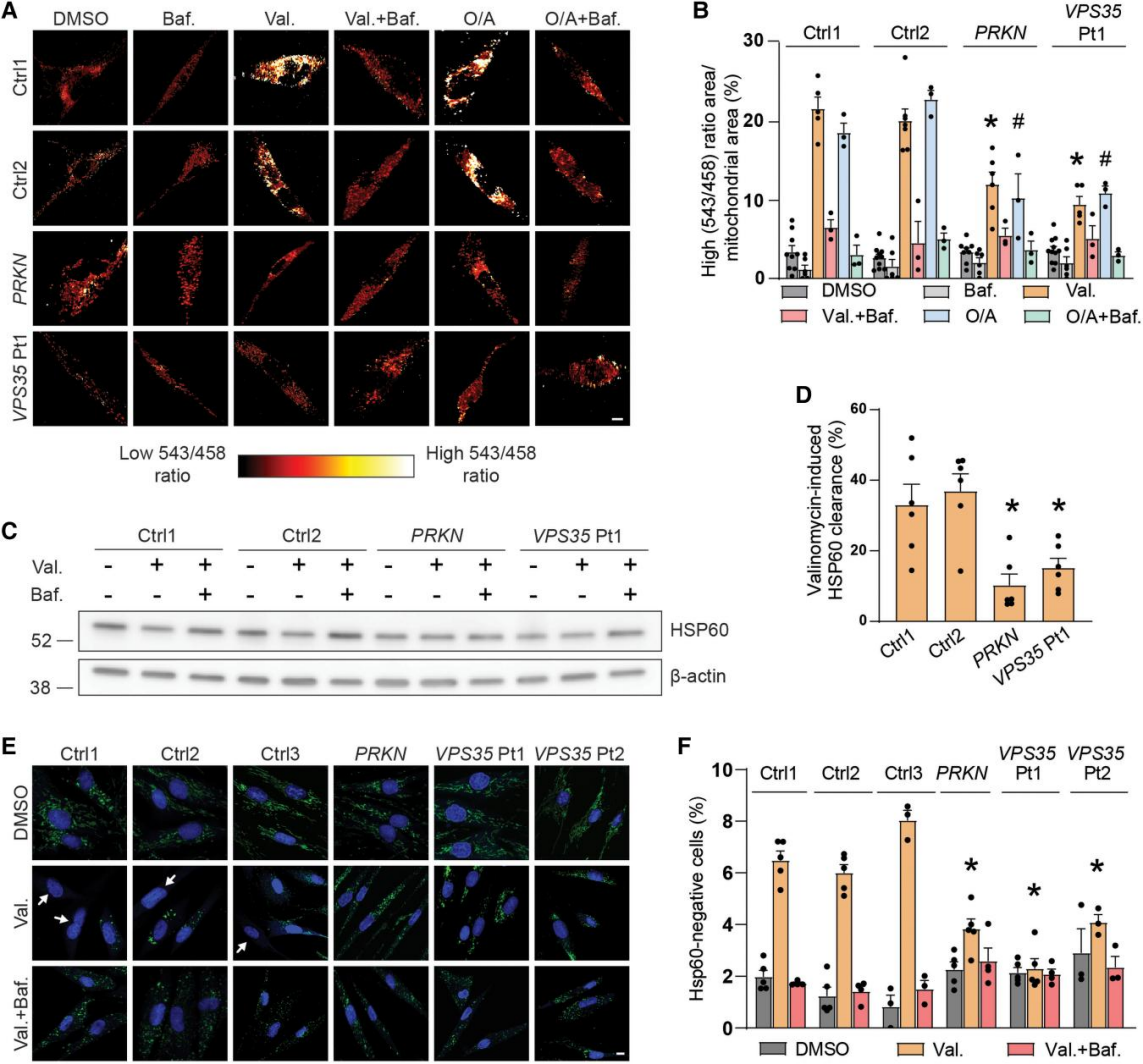
**Mitophagy is impaired in *VPS35* mutant fibroblasts.** (**A** and **B**) Control (Ctrl1, Ctrl2), *PRKN* mutant and *VPS35* patient 1 (*VPS35* Pt1) fibroblasts were transfected with mito-Keima and treated with DMSO, valinomycin (Val., 1 µM), a combination of oligomycin (10 µM) and antimycin A (4 µM) (O/A) and/or bafilomycin A1 (Baf., 100 nM) for 48 h followed by live ratiometric mito-Keima imaging. High 543/458 ratio mito-Keima signal indicates presence of mito-Keima inside lysosomes. (**B**) High mito-Keima (543/458) ratio area/total mitochondrial area was quantified as an index of mitophagy (*n* = 3–11, with approximately 15 cells analysed per experiment for each condition). *P < 0.0001 compared to Ctrl1 Val. and Ctrl2 Val. ^#^*P* < 0.05 compared to Ctrl1 O/A and Ctrl2 O/A. (**C** and **D**) Fibroblasts were treated with DMSO, Val. (1 µM) and Baf. (100 nM) for 48 h, followed by western blot for endogenous mitochondrial matrix protein HSP60. (**D**) Quantification (*n* = 6). *P < 0.05 compared to Ctrl1 and Ctrl2. (**E** and **F**) Fibroblasts from controls (Ctrl1, Ctrl2, Ctrl3) and two patients with *VPS35* mutation (*VPS35* Pt1, *VPS35* Pt2) were treated with DMSO, Val. (1 µM) and Baf. (100 nM) for 48 h, followed by immunostaining for HSP60. Nuclei were stained with TOTO-3 (blue). Arrows indicate examples of cells without detectable HSP60 staining. (**F**) Quantification (*n* = 3–5, with at least 100 cells analysed per experiment for each condition). *P < 0.05 compared to Ctrl1 Val., Ctrl2 Val. and Ctrl3 Val. Scale bars = 10 µm.

We assessed mitophagy in cultured skin fibroblasts from *VPS35* patient 1 and from two healthy controls of similar age (Ctrl1, a 68-year-old male, and Ctrl2, a 57-year-old female). In addition, we included fibroblasts from a 46-year-old female Parkinson’s disease patient with compound heterozygous *PRKN* mutations as a positive control for disrupted PINK1/parkin-mediated mitophagy in our assays, as we previously showed that PINK1/parkin-mediated mitophagy is defective in fibroblasts from this patient.^8^ We induced mitophagy by treating fibroblasts with the mitochondrial uncoupler valinomycin. We previously demonstrated that valinomycin-induced mitophagy in human skin fibroblasts depends on PINK1, parkin and DJ-1.^8,13,33^ Live imaging with the fluorescent mitophagy reporter mito-Keima showed that valinomycin triggered mitophagy in control fibroblasts and that this was suppressed by the lysosomal vacuolar-type H^+^-ATPase inhibitor bafilomycin A1, in line with previous data^8,13^ (Fig. 2A and B). Valinomycin-induced mitophagy was defective in *PRKN* mutant cells, as expected,^8^ but, interestingly, also in the *VPS35* mutant cells (Fig. 2A and B). We confirmed the mitophagy defect in the *VPS35* mutant fibroblasts by measuring valinomycin-induced clearance of the mitochondrial matrix protein HSP60 using western blot (Fig. 2C and D) and immunostaining (Fig. 2E and F). Mitophagy induced by combined exposure to electron transport chain complex III inhibitor antimycin A and complex V inhibitor oligomycin^13^ was also severely suppressed by the *VPS35* mutation (Fig. 2A and B).

We also quantified valinomycin-induced mitophagy in fibroblasts from a second, unrelated, previously described 76-year-old male Parkinson’s disease patient with a heterozygous p.D620N *VPS35* mutation (*VPS35* patient 2) and clinical onset at 59 years,^25,26^ and compared this with fibroblasts from an additional 74-year-old male healthy control (Ctrl3). *VPS35* patient 2 fibroblasts also showed a mitophagy defect (Fig. 2E and F).

**Figure 3:**
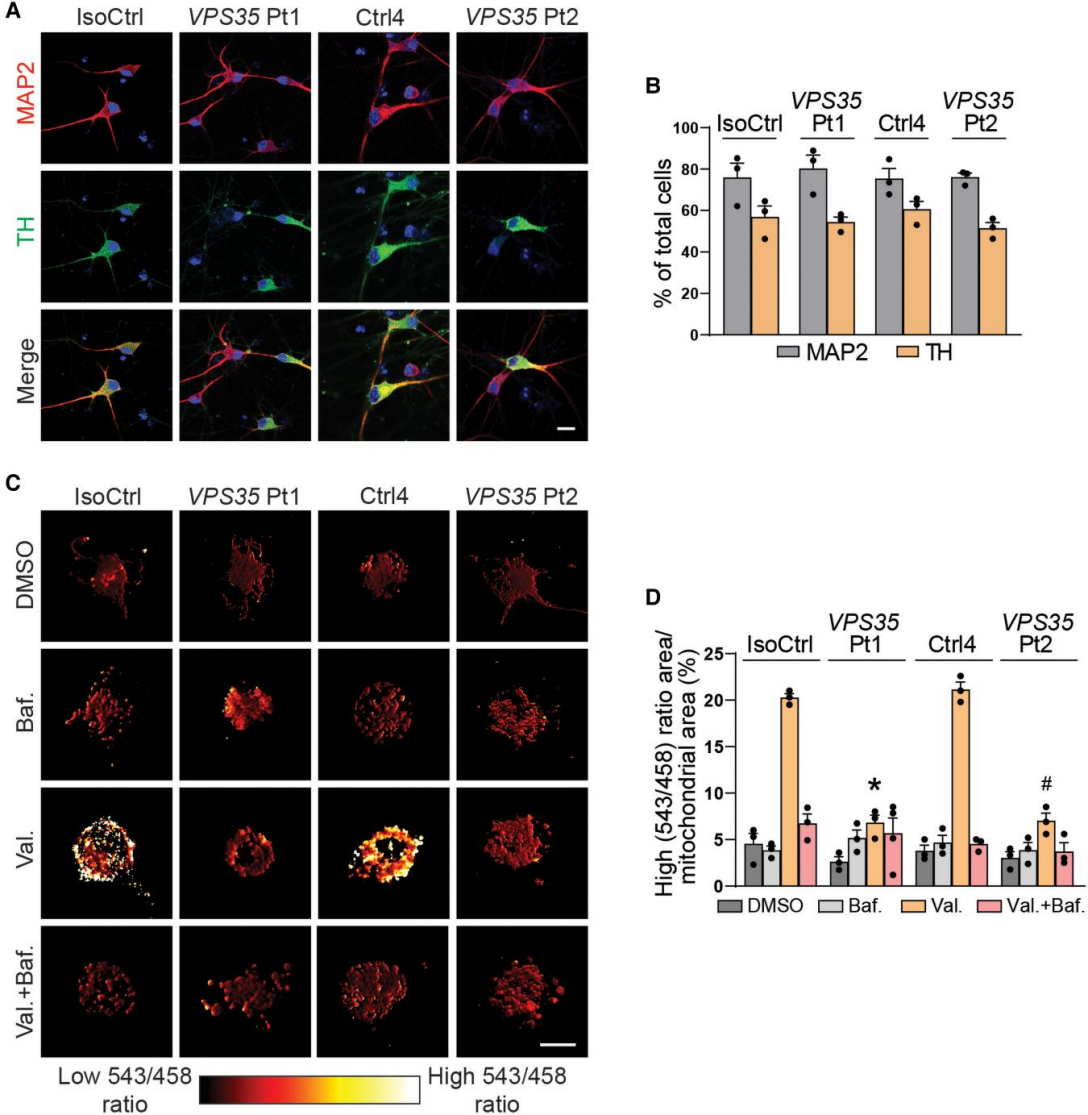
**Mitophagy is impaired in *VPS35* mutant neurons.** (**A** and **B**) On Day 50 after neuronal induction, neuronal cultures derived from induced pluripotent stem cells (iPSCs) from two patients with *VPS35* mutation (*VPS35* Pt1, *VPS35* Pt2), isogenic control iPSCs for *VPS35* Pt1 (IsoCtrl) and non-isogenic control iPSCs (Ctrl4) were immunostained for MAP2 and tyrosine hydroxylase (TH). Nuclei were stained with TOTO-3 (blue). (**B**) Quantification of percentage of TH- and MAP2-positive cells (*n* = 3, with at least 100 cells analysed per experiment for each condition). (**C** and **D**) On Day 48 after neuronal induction, iPSC-derived neurons were transduced with mito-Keima lentivirus. After 48 h, cells were treated for 24 h with DMSO, valinomycin (Val., 1 µM) and/or bafilomycin A1 (Baf., 100 nM), followed by live mito-Keima imaging. (**D**) High (543/458) ratio area/total mitochondrial area was quantified as an index of mitophagy (*n* = 3–4, with approximately 15 cells analysed per experiment for each condition). *P < 0.0001 compared to IsoCtrl Val. ^#^*P* < 0.0001 compared to Ctrl4 Val. Scale bars = 10 µm.

To also determine the effect of the *VPS35* mutation on mitophagy in human neurons, we reprogrammed the fibroblasts from *VPS35* patient 1 to iPSCs and generated a gene-corrected, isogenic control iPSC line (IsoCtrl). In addition, we used previously generated iPSCs from *VPS35* patient 2^25^ and also generated iPSCs from fibroblasts from a 68-year-old male healthy control (Ctrl4) as non-isogenic control for *VPS35* patient 2. We differentiated the iPSCs to dopaminergic neurons, as previously described.^8,29^ On Day 50 after neuronal induction, ∼80% of all cells were neurons based on immunostaining for the neuronal marker MAP2, and ∼55% of all cells were dopaminergic based on staining for tyrosine hydroxylase (TH) (Fig. 3A and B). There were no significant differences in differentiation efficiency between *VPS35* mutant and control cultures (Fig. 3B). We previously showed that valinomycin treatment of wild-type iPSC-derived neurons induces parkin-dependent mitophagy.^8^ We used lentivirus to transduce iPSC-derived neurons with mito-Keima and, importantly, found that *VPS35* mutant neurons from each of the two patients had a severe defect in valinomycin-induced mitophagy, similar to the *VPS35* fibroblasts (Fig. 3C and D).

### *VPS35* mutation impairs optineurin recruitment to depolarized mitochondria downstream of PINK1/parkin activation

**Figure 4:**
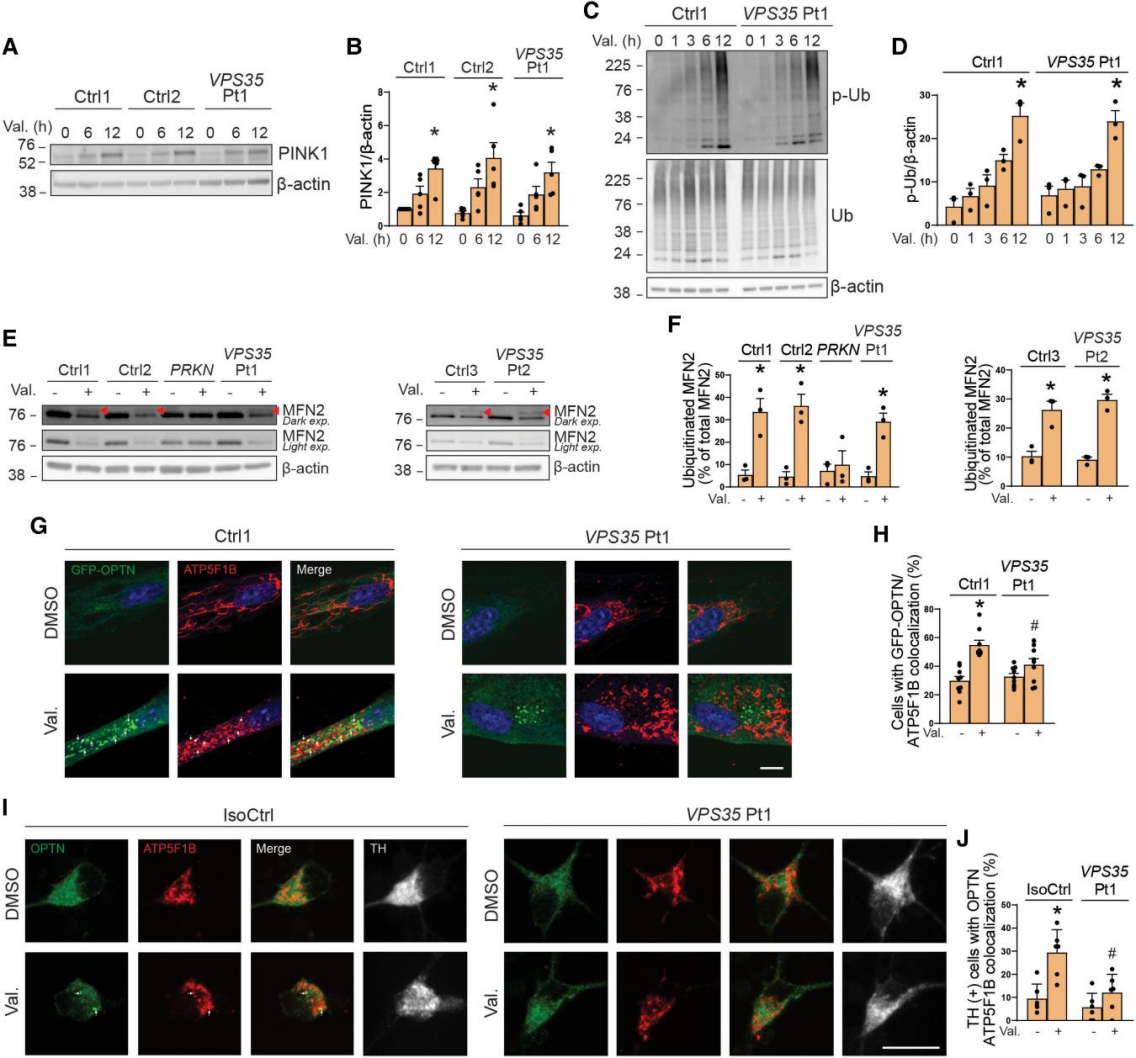
***VPS35* mutation does not affect PINK1 and parkin activation, but impairs mitochondrial recruitment of optineurin.** (**A** and **B**) Healthy control (Ctrl1, Ctrl2) and *VPS35* patient 1 (*VPS35* Pt1) fibroblasts were treated with valinomycin (Val., 1 µM) for the indicated time, followed by western blot for endogenous PINK1. (**B**) Quantification of PINK1/β-actin (normalized to Ctrl1 0 h Val. condition) (*n* = 5). *P < 0.05 compared to 0 h Val. condition in the same subject. (**C** and **D**) Fibroblasts were treated with Val. (1 µM) for the indicated time, followed by western blot for endogenous phospho-ubiquitin (p-Ub) and total ubiquitin (Ub). (**D**) Quantification of p-Ub/β-actin (*n* = 3). *P < 0.01 compared to Val. 0 h, Val. 1 h and Val. 3 h conditions in the same subject. (**E** and **F**) Fibroblasts from controls (Ctrl1, Ctrl2, Ctrl3) and two patients with *VPS35* mutation (*VPS35* Pt1, *VPS35* Pt2) were treated with DMSO or Val. (1 µM) for 3 h, followed by western blot for endogenous mitofusin 2 (MFN2). The same MFN2 blots are shown after dark and light exposure (exp.). *Red arrowheads* indicate ubiquitinated MFN2. (**F**) Quantification (*n* = 3). *P < 0.05 compared to DMSO in the same subject. (**G** and **H**) Fibroblasts were transfected with GFP-tagged optineurin (GFP-OPTN) and treated with DMSO or Val. (1 µM) for 6 h, followed by immunostaining for mitochondrial marker ATP5F1B. Nuclei were stained with TOTO-3 (blue). Arrowheads indicate GFP-OPTN puncta on mitochondria. (**H**) Quantification of percentage of cells showing GFP-OPTN/ATP5F1B colocalization (*n* = 9, with at least 50 cells analysed per experiment for each condition). *P < 0.0001 compared to Ctrl1 DMSO. ^#^*P* < 0.05 compared to Ctrl1 Val. (**I** and **J**) *VPS35* Pt1 and isogenic control (IsoCtrl) neurons were treated with DMSO or Val. (1 µM) for 6 h, followed by immunostaining for endogenous OPTN, ATP5F1B and tyrosine hydroxylase (TH). Arrowheads indicate OPTN puncta on mitochondria. (**J**) Quantification of percentage of TH-positive cells showing OPTN/ATP5F1B colocalization (*n* = 6, with at least 25 TH-positive cells analysed per experiment for each condition). *P < 0.005 compared to IsoCtrl DMSO. ^#^*P* < 0.005 compared to IsoCtrl Val. Scale bars = 10 µm.

Next, we asked which step of the PINK1/parkin-mediated mitophagy pathway was disrupted by the *VPS35* mutation. The first step in this pathway is PINK1 accumulation on the OMM due to interruption of the import and degradation of PINK1 in depolarized mitochondria.^6,7^ Accumulation of endogenous PINK1 after valinomycin treatment did not differ between *VPS35* mutant and control fibroblasts (Fig. 4A and B). Also, valinomycin-induced formation of phospho-ubiquitin, a reaction product of the PINK1 kinase, was unaffected by the *VPS35* mutation (Fig. 4C and D).

We then examined PINK1-mediated activation of parkin at the OMM of depolarized mitochondria using a MFN2 ubiquitination assay. MFN2 is an OMM protein that is ubiquitinated by activated parkin after mitochondrial depolarization and then degraded by the proteasome.^34^ Valinomycin treatment for 3 h in control fibroblasts expressing endogenous parkin resulted in ubiquitination and degradation of endogenous MFN2 (Fig. 4E and F). This was abrogated in *PRKN* mutant fibroblasts, as expected,^13^ but was intact in *VPS35* patient 1 and 2 cells (Fig. 4E and F), indicating that parkin activation at the OMM of depolarized mitochondria was preserved. However, recruitment of the GFP-tagged autophagy receptor optineurin to damaged mitochondria was significantly impaired in *VPS35* mutant fibroblasts (Fig. 4G and H). Recruitment of endogenous optineurin to depolarized mitochondria in *VPS35* mutant dopaminergic neurons was also defective (Fig. 4I and J).

**Figure 5:**
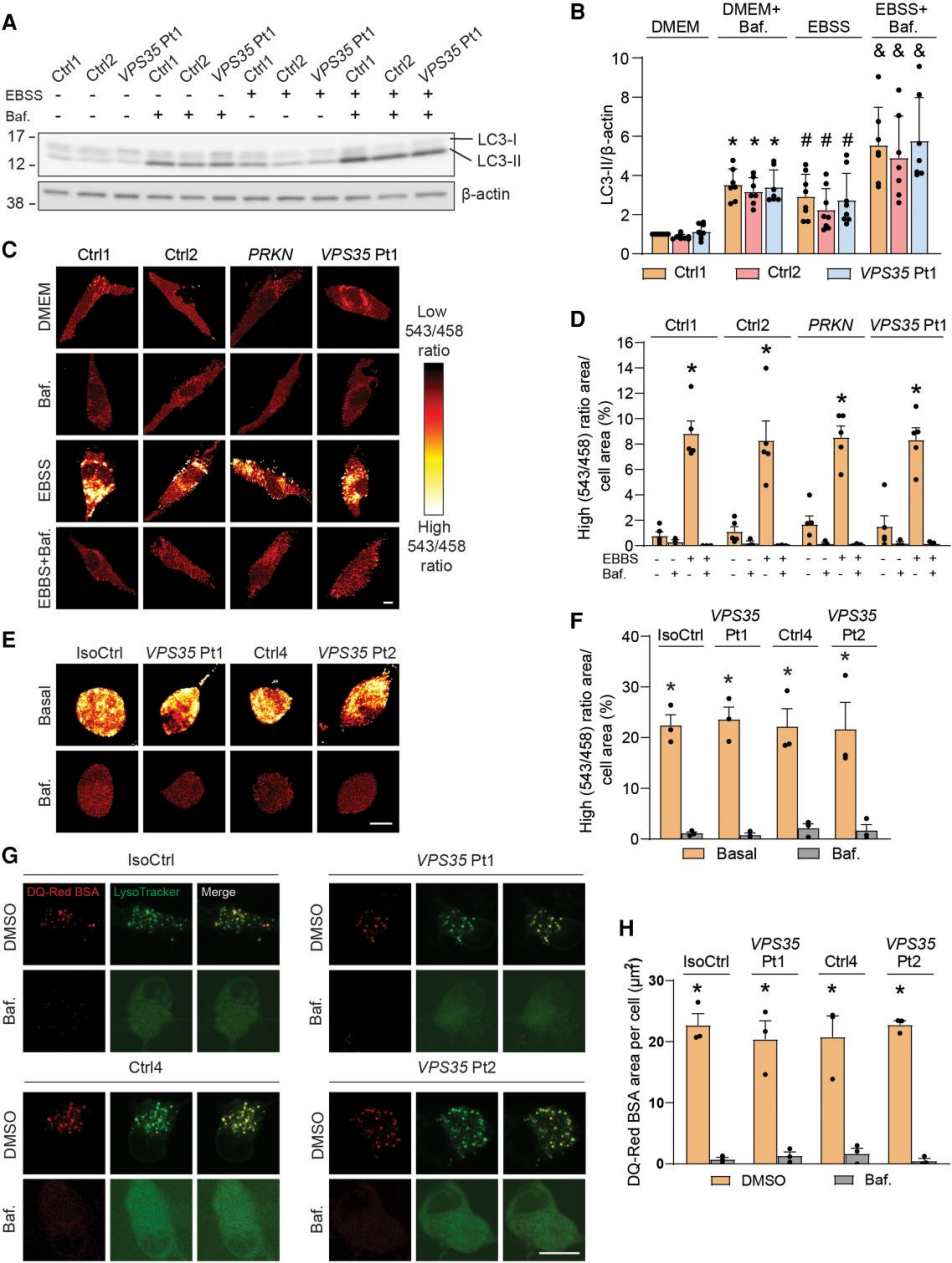
**Non-selective autophagy and lysosomal degradative capacity are preserved in *VPS35* mutant cells.** (**A** and **B**) Control (Ctrl1, Ctrl2) and *VPS35* patient 1 (*VPS35* Pt1) fibroblasts were incubated either in standard culture medium (DMEM) or in Earle’s balanced salt solution (EBSS) for 4 h to trigger starvation-induced autophagy, in the presence or absence of bafilomycin A1 (Baf., 100 nM), followed by western blot for endogenous LC3. (**B**) Quantification of LC3-II/β-actin (normalized to Ctrl1 DMEM) (*n* = 7–8). *P < 0.0001 compared to DMEM in the same subject. ^#^*P* < 0.05 compared to DMEM in the same subject. ^&^*P* < 0.05 compared to EBSS in the same subject. (**C** and **D**) Fibroblasts were transfected with Keima and incubated for 4 h in DMEM or EBSS in the presence or absence of Baf. (100 nM), followed by live Keima imaging. High (543/458) ratio signal corresponds to Keima present inside lysosomes. (**D**) High Keima (543/458) ratio area/total cell area was quantified as an index of autophagy (*n* = 3–5, with approximately 15 cells analysed per experiment for each condition). *P < 0.0001 compared to all other conditions in the same subject. (**E** and **F**) Neuronal cultures derived from iPSCs from two patients with *VPS35* mutation (*VPS35* Pt1, *VPS35* Pt2), isogenic control induced pluripotent stem cells (iPSCs) for *VPS35* Pt1 (IsoCtrl) and non-isogenic control iPSCs (Ctrl4) were transduced with Keima lentivirus and treated with DMSO or Baf. (100 nM) for 24 h, followed by live Keima imaging. (**F**) Quantification (*n* = 3, with approximately 15 cells analysed per experiment for each condition). *P < 0.01 compared to Baf. in the same subject. (**G** and **H**) *VPS35* Pt1, *VPS35* Pt2, IsoCtrl and Ctrl4 neurons were treated with DMSO or Baf. (100 nM) for 24 h before live imaging. DQ-Red BSA was added to the cells for 2 h before live imaging, and the lysosomal dye LysoTracker Green was added just before imaging. (**H**) Quantification of the area of DQ-Red BSA signal per cell (*n* = 3, with at least 30 cells analysed per experiment for each condition). *P < 0.0001 compared to Baf. in the same subject. Scale bars = 10 μm. DMEM = Dulbecco's modified Eagle's medium.

The *VPS35* mutation has been reported to impair trafficking of the lysosomal protease cathepsin D in patient fibroblasts.^35^ We therefore assessed whether the *VPS35* mutation, in addition to inhibiting mitochondrial optineurin recruitment and PINK1/parkin-mediated mitophagy, might also cause a general autophagic or lysosomal defect. We measured basal levels of lipidated LC3 (LC3-II), an autophagosome marker whose level correlates with autophagosome numbers, but found no difference between *VPS35* mutant and control fibroblasts (Fig. 5A and B). Treatment with bafilomycin A1 to block autophagosome degradation induced a similar increase in LC3-II levels in *VPS35* mutant and control fibroblasts (Fig. 5A and B), arguing against a major basal defect in lysosomal function in the mutant cells. Moreover, amino acid starvation, a trigger for non-selective autophagy that does not require autophagy receptors,^36^ induced a similar upregulation of LC3-II levels in *VPS35* and control fibroblasts, with a similar further LC3-II increase when bafilomycin A1 was added (Fig. 5A and B). We also measured starvation-induced autophagy using live imaging with Keima, i.e. the same probe as mito-Keima but without the mitochondrial targeting sequence,^8,13,31^ and again found no difference between *VPS35* and control fibroblasts (Fig. 5C and D). Also, Keima imaging showed no difference in basal general autophagy flux levels between *VPS35* mutant and control neurons (Fig. 5E and F).

To assess lysosomal degradative capacity, we used a DQ-Red BSA assay. DQ-Red BSA is a bovine serum albumin derivative conjugated to a self-quenched fluorophore that is taken up via endocytosis and targeted to the lysosome and becomes fluorescent upon proteolysis in the lysosome. We found no significant difference in the area of DQ-Red fluorescent signal between neurons from the *VPS35* patients and isogenic and non-isogenic control neurons (Fig. 5G and H).

In summary, the *VPS35* mutation caused a deficit of PINK1/parkin-mediated mitophagy and mitochondrial optineurin recruitment, but no general autophagy defect.

### *VPS35* knockdown rescues mitophagy in *VPS35* mutant cells

**Figure 6:**
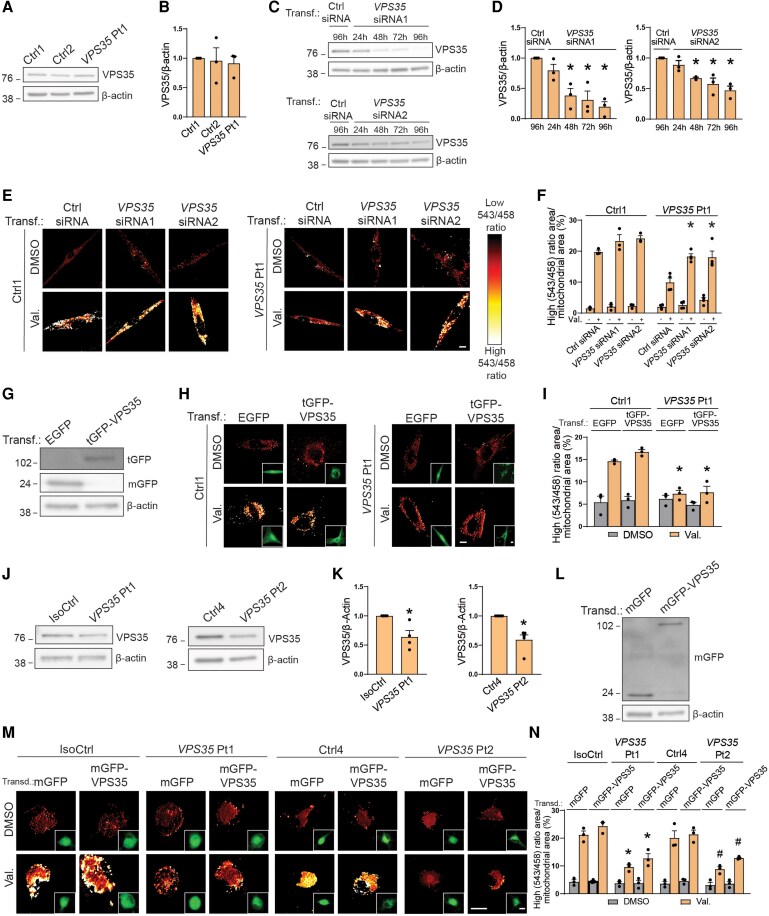
***VPS35* knockdown rescues mitophagy in *VPS35* mutant cells.** (**A** and **B**) Western blot for endogenous VPS35 in fibroblasts from healthy controls (Ctrl1, Ctrl2) and *VPS35* patient 1 (*VPS35* Pt1). (**B**) Quantification of VPS35/β-actin (normalized to Ctrl1) (*n* = 3). (**C** and **D**) Control (Ctrl1) fibroblasts were transfected (Transf.) with control (Ctrl) siRNA, *VPS35* siRNA1 or *VPS35* siRNA2. Western blot was performed at the indicated time points after transfection. (**D**) Quantification of VPS35/β-actin (normalized to Ctrl siRNA) (*n* = 3). *P < 0.05 compared to Ctrl siRNA. (**E** and **F**) Fibroblasts were transfected with mito-Keima and indicated siRNAs. After 72 h, cells were treated with DMSO or valinomycin (Val., 1 µM) for 48 h, followed by mito-Keima imaging. (**F**) Quantification (*n* = 3–4, with approximately 15 cells analysed per experiment for each condition). *P < 0.001 compared to Val. Ctrl siRNA condition in *VPS35* mutant cells. (**G**) Control (Ctrl1) fibroblasts were transfected with EGFP or tGFP-VPS35, followed by western blot with anti-tGFP (which recognizes tGFP but not EGFP or mGFP) and anti-mGFP (which recognizes mGFP and EGFP but not tGFP) antibodies. (**H** and **I**) Ctrl1 and *VPS35* mutant fibroblasts were co-transfected with mito-Keima and either EGFP or tGFP-VPS35. After 24 h, cells were treated with DMSO or Val. (1 µM) for 48 h, followed by mito-Keima imaging. *Insets* in **H** show that the imaged cells are GFP-positive. (**I**) Quantification (*n* = 3, with approximately 15 cells analysed per experiment for each condition). *P < 0.001 compared to EGFP Val. in Ctrl1 cells and to tGFP-VPS35 Val. in Ctrl1 cells. (**J** and **K**) Western blot for endogenous VPS35 in neuronal cultures derived from induced pluripotent stem cells (iPSCs) from two patients with *VPS35* mutation (*VPS35* Pt1, *VPS35* Pt2), isogenic control iPSCs for *VPS35* Pt1 (IsoCtrl) and non-isogenic control iPSCs (Ctrl4). (**K**) Quantification of VPS35/β-actin (normalized to respective control) (*n* = 4–5). *P < 0.05 compared to control. (**L**) Control (IsoCtrl) iPSC-derived neurons were transduced with lentivirus expressing either mGFP or mGFP-VPS35, followed by western blot with anti-mGFP antibody. (**M** and **N**) On Day 46 after neuronal induction, *VPS35* Pt1, *VPS35* Pt2, IsoCtrl and Ctrl4 neurons were transduced (Transd.) with lentivirus expressing either mGFP or mGFP-VPS35. After 24 h, neurons were transduced with mito-Keima lentivirus. On Day 49, neurons were treated with DMSO or Val. (1 µM) for 24 h. Mito-Keima imaging was performed on Day 50. *Insets* in **M** show that the imaged cells are GFP-positive. (**N**) Quantification (*n* = 3, with approximately 15 cells analysed per experiment for each condition). *P < 0.001 compared to Val. mGFP condition in IsoCtrl and to Val. mGFP-VPS35 condition in IsoCtrl. ^#^*P* < 0.05 compared to Val. mGFP condition in Ctrl4 and to Val. mGFP-VPS35 condition in Ctrl4. Scale bars = 10 μm.

Next, we determined whether the mitophagy defect of *VPS35* mutant cells was due to loss or gain of VPS35 function. Endogenous VPS35 protein levels were similar in *VPS35* mutant and wild-type fibroblasts (Fig. 6A and B), consistent with a previous study.^37^ Interestingly, *VPS35* knockdown using different siRNAs (Fig. 6C and D) rescued mitophagy in *VPS35* mutant fibroblasts (Fig. 6E and F), while overexpression of wild-type VPS35 did not (Fig. 6G–I), strongly suggesting that a gain-of-function of the mutant VPS35 protein caused the mitophagy defect.

To our surprise, endogenous VPS35 protein levels in iPSC-derived neurons from *VPS35* patients 1 and 2 were only ∼60% of the levels in the respective isogenic and non-isogenic control neurons (Fig. 6J and K). Nevertheless, overexpression of wild-type VPS35 failed to rescue the mitophagy defect of *VPS35* patients 1 and 2 neurons despite successful transduction (Fig. 6L–N), arguing against loss of VPS35 function as the mechanism underlying mitophagy disruption.

### LRRK2 kinase inhibition and PPM1H overexpression rescue mitophagy in *VPS35* mutant cells

**Figure 7:**
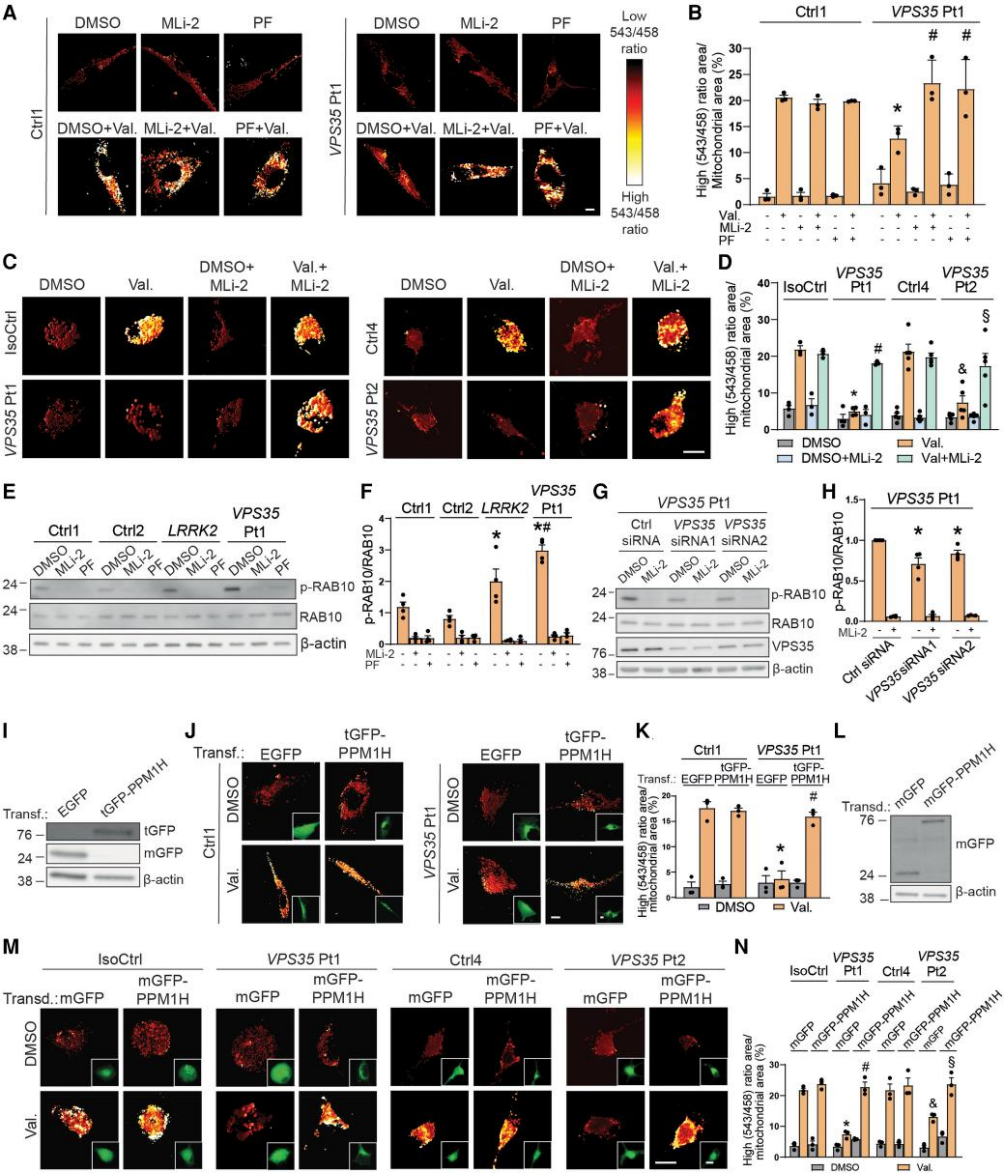
**LRRK2 kinase inhibition and PPM1H overexpression rescue mitophagy in *VPS35* mutant cells.** (**A** and **B)** Control (Ctrl1) and *VPS35* patient 1 fibroblasts were transfected with mito-Keima and treated with DMSO or LRRK2 kinase inhibitors MLi-2 (150 nM) or PF-06447475 (PF, 0.5 µM) for 24 h, followed by treatment with DMSO or valinomycin (Val., 1 µM) for 48 h in the continued presence or absence of MLi-2 or PF and live mito-Keima imaging. (**B**) Quantification (*n* = 3, with approximately 15 cells analysed per experiment for each condition). *P < 0.05 compared to Ctrl1 Val. without MLi-2 or PF. ^#^*P* < 0.01 compared to *VPS35* Val. without MLi-2 or PF. (**C** and **D**) On Day 47 after neuronal induction, neurons derived from induced pluripotent stem cells (iPSCs) from two patients with *VPS35* mutation (*VPS35* Pt1, *VPS35* Pt2), isogenic control iPSCs for *VPS35* Pt1 (IsoCtrl) and non-isogenic control iPSCs (Ctrl4) were transduced with mito-Keima lentivirus. On Day 48, neurons were treated with DMSO or MLi-2 (150 nM) for 24 h, followed by 24 h treatment with DMSO or Val. (1 µM) in the continued presence or absence of MLi-2. Mito-Keima imaging was performed on Day 50. (**D**) Quantification (*n* = 3–5, with approx. 15 cells analysed per experiment for each condition). *P < 0.0001 compared to IsoCtrl. Val. ^#^*P* < 0.0001 compared to *VPS35* Pt1 Val. ^&^*P* < 0.0001 compared to Ctrl4 Val. ^§^*P* < 0.01 compared to *VPS35* Pt2 Val. (**E** and **F**) Control (Ctrl1, Ctrl2), p.R1441C *LRRK2* mutant and *VPS35* patient 1 fibroblasts were treated with DMSO or LRRK2 kinase inhibitors MLi-2 (150 nM) or PF-06447475 (PF, 0.5 µM) for 24 h, followed by western blot for endogenous phospo-RAB10 (p-RAB10) and total RAB10. (**F**) Quantification of p-RAB10/total RAB10 (*n* = 4). *P < 0.05 compared to Ctrl1 DMSO and Ctrl2 DMSO. ^#^*P* < 0.01 compared to *LRRK2* DMSO. (**G** and **H**) *VPS35* patient 1 fibroblasts were transfected with Ctrl siRNA, *VPS35* siRNA1 or *VPS35* siRNA2. After 72 h, fibroblasts were treated with DMSO or MLi-2 (150 nM) for 24 h, followed by western blot for endogenous p-RAB10, total RAB10 and VPS35. (**H**) Quantification of p-RAB10/total RAB10 (normalized to the DMSO Ctrl siRNA condition) (*n* = 4). *P < 0.05 compared to DMSO Ctrl siRNA. (**I**) Control (Ctrl1) fibroblasts were transfected with EGFP or tGFP-PPM1H, followed by western blot with anti-tGFP (which recognizes tGFP but not EGFP or mGFP) and anti-mGFP (which recognizes mGFP and EGFP but not tGFP) antibodies. (**J** and **K**) Control (Ctrl1) and *VPS35* patient 1 fibroblasts were co-transfected (Transf.) with mito-Keima and either EGFP or tGFP-PPM1H. After 24 h, cells were treated with DMSO or Val. (1 µM) for 48 h, followed by mito-Keima imaging. (**K**) Quantification (*n* = 3, with approx. 15 cells analysed per experiment for each condition). *P < 0.0001 compared to Val. EGFP condition in Ctrl1 and to Val. tGFP-PPM1H condition in Ctrl1. ^#^*P* < 0.0001 compared to Val. EGFP condition in *VPS35* mutant cells. (**L**) Control (IsoCtrl) iPSC-derived neurons were transduced with lentivirus expressing either mGFP or mGFP-PPM1H, followed by western blot with anti-mGFP antibody. (**M** and **N**) On Day 46 after neuronal induction, *VPS35* Pt1, *VPS35* Pt2, IsoCtrl and Ctrl4 neurons were transduced (Transd.) with lentivirus expressing either mGFP or mGFP-PPM1H. After 24 h, neurons were transduced with mito-Keima lentivirus. On Day 49, neurons were treated with DMSO or Val. (1 µM) for 24 h. Mito-Keima imaging was performed on Day 50. (**N**) Quantification (*n* = 3, with approx. 15 cells analysed per experiment for each condition). *P < 0.0001 compared to Val. mGFP condition in IsoCtrl and to Val. mGFP-PPM1H condition in IsoCtrl. ^#^*P* < 0.0001 compared to Val. mGFP condition in *VPS35* Pt1 neurons. ^&^*P* < 0.05 compared to Val. mGFP condition in Ctrl4 and to Val. mGFP-PPM1H condition in Ctrl4. ^§^*P* < 0.01 compared to Val. mGFP condition in *VPS35* Pt2 neurons. Scale bars = 10 μm.

We wondered which gain-of-function of the mutant VPS35 protein could underlie the observed mitophagy defect. One candidate mechanism could be stimulation of LRRK2 kinase activity by mutant VPS35. Mir *et al*.^24^ reported that LRRK2 kinase activity is enhanced in embryonic fibroblasts and various tissues from VPS35 D620N knock-in mice and in neutrophils and monocytes from Parkinson’s disease patients with this mutation, and *LRRK2* mutations were found to inhibit PINK1/parkin-mediated mitophagy through increased LRRK2 kinase activity.^10,13^ In addition, the pattern of the mitophagy defect in *VPS35* mutant fibroblasts (i.e. intact PINK1 and parkin activation and impaired optineurin recruitment in the absence of a non-selective autophagy defect) closely resembled what was previously observed in *LRRK2* mutant fibroblasts,^13^ consistent with the hypothesis that the *VPS35* mutation could disrupt mitophagy via the LRRK2 kinase. Indeed, treatment with two different LRRK2 kinase inhibitors (MLi-2 and PF-06447475) fully rescued the mitophagy defect of *VPS35* mutant fibroblasts (Fig. 7A and B). Moreover, LRRK2 kinase inhibition with MLi-2 also completely reversed the mitophagy defect of *VPS35* patient 1 and 2 neurons (Fig. 7C and D). Taken together, this suggested that increased LRRK2 kinase activity mediated the mitophagy defect of *VPS35* mutant fibroblasts and neurons.

To assess LRRK2 kinase activity in the *VPS35* mutant fibroblasts, we measured levels of the LRRK2 substrate RAB10 phosphorylated at T73 (p-RAB10) by immunoblotting. Levels of p-RAB10 were strikingly elevated in the *VPS35* mutant cells, even more so than in fibroblasts from a Parkinson’s disease patient with the p.R1441C mutation in *LRRK2* (Fig. 7E and F), consistent with previous work.^24^  *VPS35* knockdown in *VPS35* mutant fibroblasts significantly reduced p-RAB10 levels (Fig. 7G and H), suggesting that a gain-of-function of mutant VPS35 led to increased LRRK2 kinase activity.

To test whether increased phosphorylation of RAB substrates of LRRK2 was responsible for the mitophagy defect of *VPS35* mutant fibroblasts, we overexpressed PPM1H, a phosphatase that dephosphorylates RAB10 and multiple other RAB substrates of LRRK2.^38^ Importantly, PPM1H overexpression completely restored mitophagy in the *VPS35* mutant fibroblasts (Fig. 7I–K). In *VPS35* mutant iPSC-derived neurons, we were unable to detect p-RAB10 on western blot. This is in line with studies showing that p-RAB10 is hardly detectable in nervous tissue.^39,40^ Nevertheless, PPM1H overexpression also fully rescued mitophagy in neurons from *VPS35* patients 1 and 2 (Fig. 7L–N), strongly suggesting that the mitophagy defect of these cells was also driven by increased phosphorylation of one or more RAB substrates of LRRK2.

### LRRK2 and VPS35 are proximity partners in neurons

**Figure 8:**
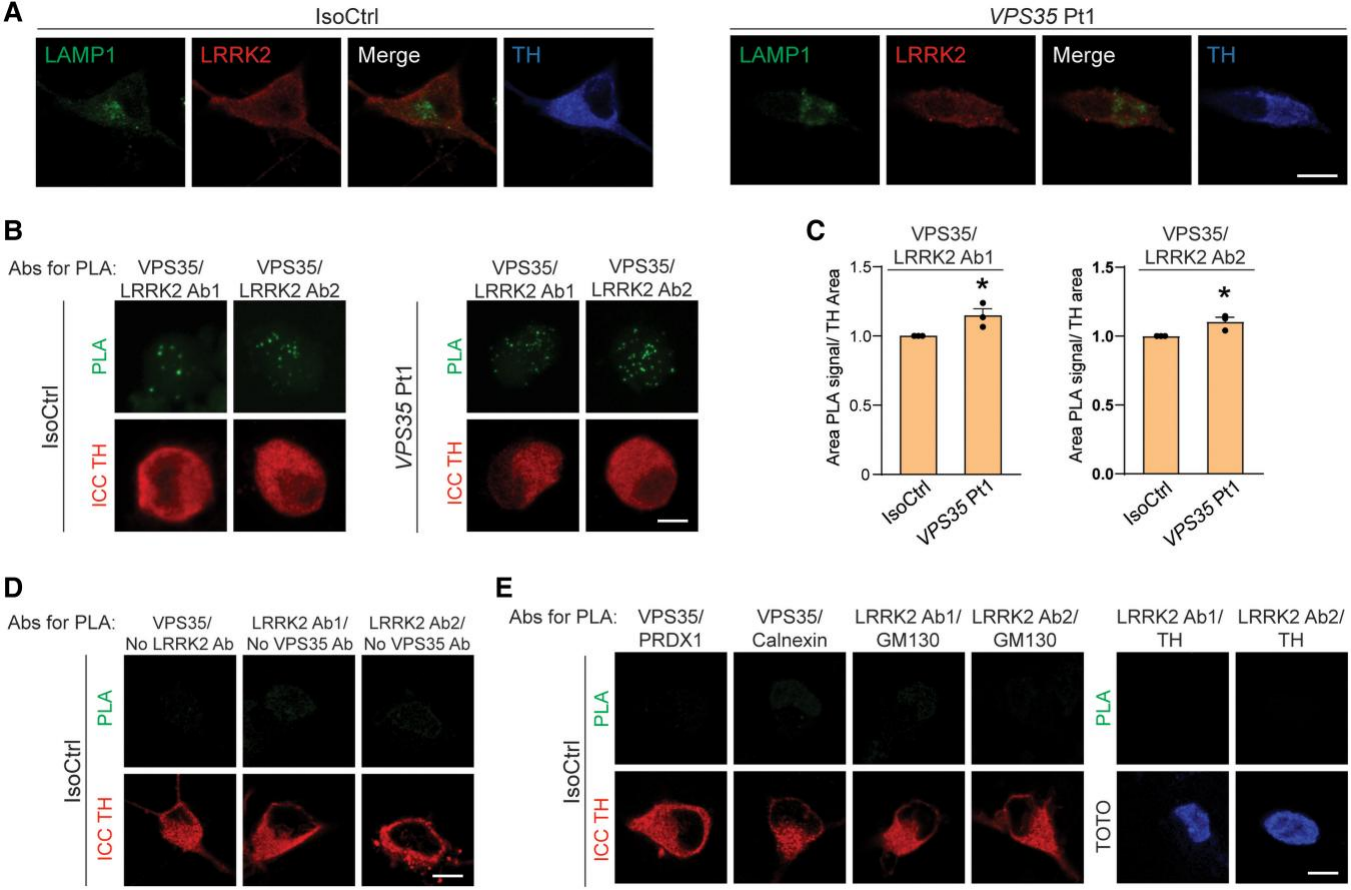
**Endogenous VPS35 and LRRK2 are proximity partners in dopaminergic neurons.** (**A**) On Day 50 after neuronal induction, iPSC-derived isogenic control (IsoCtrl) and *VPS35* patient 1 neurons were immunostained for endogenous LRRK2, the lysosomal marker LAMP1 and tyrosine hydroxylase (TH). Representative examples are shown of three experiments (approximately 25 cells analysed per experiment for each genotype). (**B** and **C**) Proximity ligation assays (PLA) with antibodies (Abs) against VPS35 and LRRK2 (LRRK2 Ab1: Abcam ab133476; LRRK2 Ab2: Abcam ab133518) combined with immunocytochemical staining (ICC) for tyrosine hydroxylase (TH) were performed on iPSC-derived isogenic control (IsoCtrl) and *VPS35* patient 1 (*VPS35* Pt1) neurons on Day 50 after neuronal induction. (**C**) Quantification of the area of VPS35/LRRK2 PLA signal divided by total TH-positive area (normalized to IsoCtrl) (*n* = 3, with at least 25 cells analysed per experiment for each condition). *P < 0.05 compared to IsoCtrl. (**D** and **E**) Negative control experiments in IsoCtrl neurons demonstrating specificity of the VPS35/LRRK2 PLA signal. (**D**) As technical negative controls, PLA experiments (combined with TH immunostaining) were performed in which either the VPS35 or the LRRK2 primary antibody was omitted. (**E**) As biological negative controls, PLA experiments were performed for either VPS35 or LRRK2 and proteins not known to interact with VPS35 or LRRK2 (PRDX1, calnexin, GM130, TH). In the *leftmost four columns*, PLA was combined with TH immunostaining. In the *rightmost two columns*, PLA was combined with TOTO-3 nuclear staining (blue). Scale bars = 10 μm. iPSC = induced pluripotent stem cell.

It has been proposed that the *VPS35* mutation may cause lysosomal stress, which then induces LRRK2 translocation to lysosomal membranes and LRRK2 hyperactivation.^41,42^ However, we could not detect colocalization of endogenous LRRK2 with lysosomes in either *VPS35* mutant or isogenic control dopaminergic neurons (Fig. 8A).

We considered the possibility that mutant VPS35 could affect LRRK2 more directly than via lysosomal stress-dependent LRRK2 activation. McLeod *et al*.^43^ reported coimmunoprecipitation of overexpressed wild-type LRRK2 with overexpressed GFP-tagged wild-type and mutant VPS35 from SHSY-5Y cell lysates and of endogenous VPS35 with endogenous LRRK2 from mouse whole brain lysates. However, to our knowledge, other studies have not found evidence of VPS35/LRRK2 interaction using biochemical methods. A possible explanation could be that only a small subset of VPS35 and LRRK2 molecules encounter each other at any given time point at a critical subcellular site, and that this signal is too diluted in whole-cell lysates to be detectable. To circumvent this, we applied *in situ* PLA. PLA yields a signal when two proteins of interest are within 40 nm of each other (which typically implies that they interact directly or indirectly), and has high spatial resolution and sensitivity.^44,45^ Interestingly, using a knockout-validated VPS35 antibody and two different knockout-validated LRRK2 antibodies, we found clearly positive PLA dots for endogenous VPS35 and LRRK2 in control iPSC-derived dopaminergic neurons (Fig. 8B). Specificity of the PLA signal was demonstrated using technical negative controls, in which we omitted either the VPS35 or the LRRK2 primary antibody (Fig. 8D), as well as biological negative controls, in which we performed PLA for VPS35 or LRRK2 and proteins not known to interact with VPS35 or LRRK2 (Fig. 8E). Taken together, these data indicated that endogenous VPS35 and LRRK2 are proximity partners at discrete sites in human control dopaminergic neurons. In *VPS35* mutant dopaminergic neurons, the area of the VPS35/LRRK2 PLA signal showed a small (∼15%) but significant increase compared with isogenic control dopaminergic neurons, suggesting that the *VPS35* mutation led to enhanced proximity of VPS35 and LRRK2 (Fig. 8B and C).

## Discussion

We show that fibroblasts and iPSC-derived dopaminergic neurons from Parkinson’s disease patients carrying an endogenous pathogenic heterozygous *VPS35* mutation have a defect in PINK1/parkin-dependent mitophagy, a pathway already known to be disrupted by recessive Parkinson’s disease gene mutations^6-8^ as well as by dominant *LRRK2* mutations.^9,10,13,17^ Moreover, the *VPS35* mutation exerts this effect on mitophagy via increased LRRK2 kinase activity, suggesting that this mutation acts upstream of LRRK2.

The dominant inheritance pattern of the *VPS35* mutation suggests that it acts either through toxic gain-of-function or through loss-of-function (via haploinsufficiency or a dominant-negative effect). Previous studies addressing these potential mechanisms have provided mixed results. The *VPS35* mutation does not seem to affect assembly of VPS35 with other retromer subunits^35,37^ or the sorting of most types of retromer cargo,^37,46^ but impairs the ability of VPS35 to bind to the WASH complex and recruit this complex to endosomes,^46,47^ suggesting that the mutation may induce loss of some but not all functions of the protein. Overexpression of human D620N VPS35 in substantia nigra of rodents produced more dopaminergic cell loss than overexpression of wild-type VPS35,^37,48^ consistent with either a toxic gain-of-function or a dominant-negative effect. By contrast, a study in *Drosophila* found no evidence of dominant toxicity from the human D620N VPS35 protein, but reported that the mutation conferred a partial loss-of-function.^49^ Here, we demonstrate that the mitophagy defect induced by the endogenous *VPS35* mutation in patient cells was fully rescued by *VPS35* knockdown, but not by overexpression of wild-type VPS35. This indicated that the defective mitophagy phenotype of the patient cells was due to a toxic gain-of-function mechanism.

Surprisingly, we found that VPS35 protein levels were ∼40% lower in *VPS35* mutant iPSC-derived neurons compared with isogenic and non-isogenic control neurons. This was unexpected, as VPS35 protein levels were found to be unaltered in *VPS35* mutant patient fibroblasts^37^ and in brains of heterozygous and homozygous *VPS35* p.D620N knock-in mice.^50,51^ It will be important to assess this in iPSC-derived neurons from additional patients with this mutation. It would also be interesting to differentiate the *VPS35* mutant iPSCs into other cell types and determine whether the reduction of VPS35 protein level is cell type-dependent. In any case, the reduction of VPS35 protein levels in human *VPS35* mutant neurons did not seem to be responsible for the observed mitophagy defect, because successful overexpression of wild-type VPS35 did not mitigate the defective mitophagy phenotype. Nevertheless, a partial loss of VPS35 protein could potentially compromise neuronal VPS35 functions that were not assayed in our study, such as the proposed role of VPS35 in synaptic vesicle recycling,^52^ which, in combination with the mitophagy defect, could contribute to neuronal damage.

LRRK2 kinase inhibitors as well as overexpression of PPM1H, a phosphatase for RAB substrates of LRRK2,^38^ completely rescued the mitophagy defect of the *VPS35* mutant fibroblasts and neurons, strongly suggesting that this defect was mediated by increased LRRK2 kinase activity towards its RAB substrates. Indeed, T73-phosphorylated levels of the LRRK2 substrate RAB10 were strongly increased in *VPS35* mutant fibroblasts, even more so than in p.R1441C *LRRK2* mutant fibroblasts, indicating that the *VPS35* mutation stimulates LRRK2 kinase activity, as first reported by Mir *et al*.^24^ We previously showed in *LRRK2* mutant patient fibroblasts that increased LRRK2-mediated phosphorylation of RAB10 interfered with binding of RAB10 to optineurin and thereby inhibited optineurin recruitment to depolarized mitochondria, downstream of intact PINK1 and parkin activation.^13^ Remarkably, the mitophagy defect of *VPS35* mutant fibroblasts showed a similar pattern of preserved PINK1 and parkin activation and impaired downstream mitochondrial optineurin recruitment, consistent with this mitophagy defect being mediated by increased LRRK2 kinase activity. In contrast to *VPS35* mutant fibroblasts, we could not detect p-RAB10 in *VPS35* mutant neurons. Previous studies have also indicated that p-RAB10 is difficult to detect in nervous tissue extracts.^39,40^ Nevertheless, PPM1H overexpression completely rescued the mitophagy defect of *VPS35* mutant neurons, strongly suggesting that this defect was also due to enhanced phosphorylation of one or more RAB substrates of LRRK2.

An important question is how the D620N VPS35 protein activates the LRRK2 kinase. According to one hypothesis, the *VPS35* mutation may impair the retromer’s retrograde cargo trafficking function, resulting in lysosomal stress. Lysosomal stress may then cause LRRK2 translocation to lysosomal membranes where LRRK2 becomes activated and hyperphosphorylates RAB substrates.^41,42^ Mass spectrometric analysis of lysosomes isolated from homozygous knock-in p.D620N *VPS35* mouse embryonic fibroblasts (MEFs) transduced with LysoTag (TMEM192-3xHA) indeed showed alterations in lysosomal protein content, and homozygous *VPS35* mutant MEFs transduced with LysoTag showed increased colocalization of overexpressed GFP-LRRK2 with lysosomes.^41^ In our study, heterozygous *VPS35* mutant human cells showed intact basal and starvation-induced autophagy and preserved lysosomal degradative capacity, arguing against the presence of a major lysosomal defect, although this does not exclude more subtle levels of lysosomal stress. Also, we could not detect increased colocalization of endogenous LRRK2 with lysosomes in heterozygous *VPS35* mutant patient dopaminergic neurons compared with isogenic control neurons. Interestingly, our PLA experiments in dopaminergic neurons revealed the intimate proximity of endogenous VPS35 and LRRK2. Moreover, the area of this PLA signal was significantly increased in *VPS35* mutant neurons compared with isogenic control neurons. This increase was relatively small (∼15%), but it should be kept in mind that these are heterozygous neurons and that total VPS35 protein levels in the mutant neurons were ∼40% lower than in controls. These data suggest that a subset of VPS35 and LRRK2 molecules encounter each other at distinct subcellular sites and that mutant VPS35 may affect LRRK2 also less indirectly than via the retromer-dependent lysosomal stress route. Future studies will need to determine the identity of these subcellular sites and to assess how the proximity of VPS35 and LRRK2 is affected by different types of neuronal stress.

The mutant VPS35-induced defect in mitophagic removal of damaged mitochondria could explain previously observed mitochondrial abnormalities in SH-SY5Y cells, patient fibroblasts and mouse and patient neurons carrying the *VPS35* mutation, such as increased mitochondrial fragmentation, reduced mitochondrial membrane potential, increased production of reactive oxygen species, decreased ATP levels and impaired mitochondrial bioenergetics.^48,53-55^ Other reported mechanisms by which VPS35 could affect mitochondria include effects on mitochondrial dynamics via interaction with DLP1 or degradation of MUL1^48,56^ and VPS35 involvement in cargo transport from mitochondria to peroxisomes via mitochondria-derived vesicles.^57^

Limitations of our study included the use of cells from only two patients with the *VPS35* mutation and only one iPSC clone per patient. Another limitation was that lysosomal degradative capacity was assessed with the DQ-Red BSA assay at only one time point and differences at other time points may have been missed. Strengths of our study were the analysis of cells expressing VPS35, LRRK2 and other relevant proteins, such as PINK1 and parkin, at endogenous levels and the comparison of *VPS35* mutant patient neurons with isogenic control neurons. To our knowledge, no other studies comparing *VPS35* mutant patient neurons with isogenic control neurons have been reported yet.

In conclusion, we show that the *VPS35* mutation impairs PINK1/parkin-mediated mitophagy via a gain-of-function mechanism that involves stimulation of LRRK2 kinase activity. Thus, dominant mutations in the *VPS35/LRRK2* axis block a pathway that critically depends on proteins encoded by the autosomal recessive Parkinson’s disease genes and that is also inhibited by increased levels of wild-type α-synuclein.^29^ It remains to be addressed whether disruption of this pathway is also a primary driver of the neuronal damage that occurs *in vivo* in Parkinson’s disease patient brains.

## Supplementary Material

awaf414_Supplementary_Data

## Data Availability

Data supporting the findings are available upon reasonable request.
