# Supplementary material for: VPS35 mutation inhibits PINK1/parkin-mediated mitophagy via increased LRRK2 kinase activity
Source: Brain. 2025 Oct 30;149(7):2363–79. doi: 10.1093/brain/awaf414 (PMC13337223; doi:10.1093/brain/awaf414)
Supplement: awaf414_Supplementary_Data [file awaf414_supplementary_data.zip › brain-2025-01360-File010.pdf]

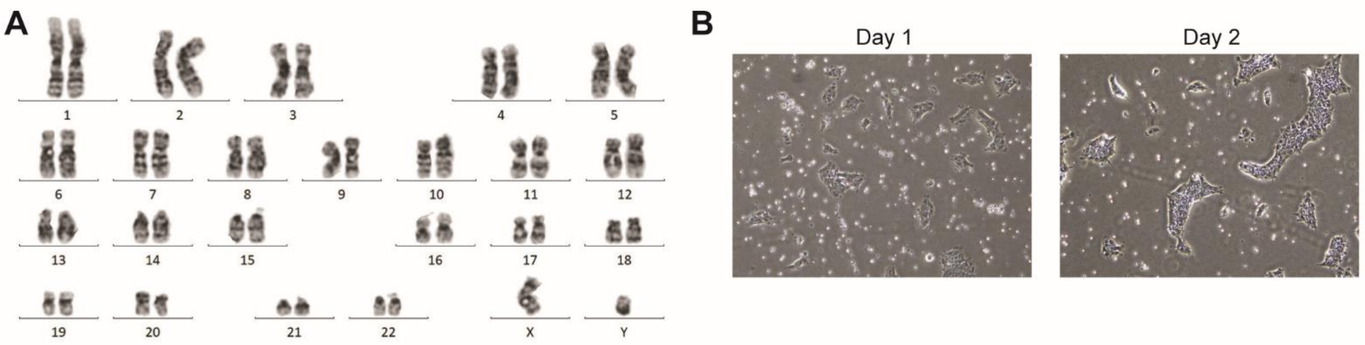

C

	VPS35 mutant fibroblasts		*VPS35* mutant iPSCs	
Marker	Allele 1	Allele 2	Allele 1	Allele 2
AMEL	X	Y	X	Y
CSF1PO	11	12	11	12
D13S317	12		12	
D16S539	11	12	11	12
D18S51	12	16	12	16
D19S433	14		14	
D21S11	30	31.2	30	31.2
D2S1338	17	24	17	24
D3S1358	15	18	15	18
D5S818	11	12	11	12
D7S820	10	11	10	11
D8S1179	13	16	13	16
FGA	23	25	23	25
TH01	6	9.3	6	9.3
TPOX	8		8	
vWA	18		18	

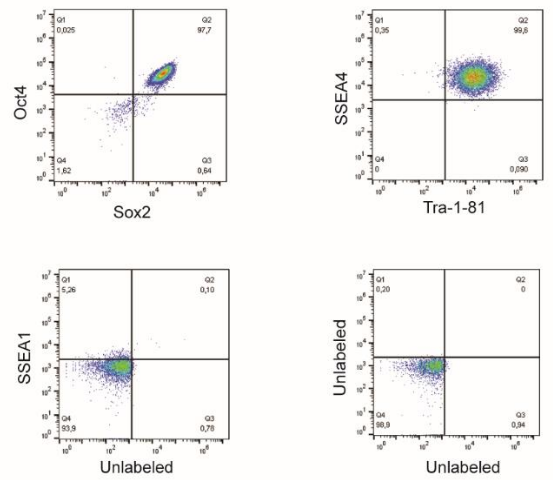

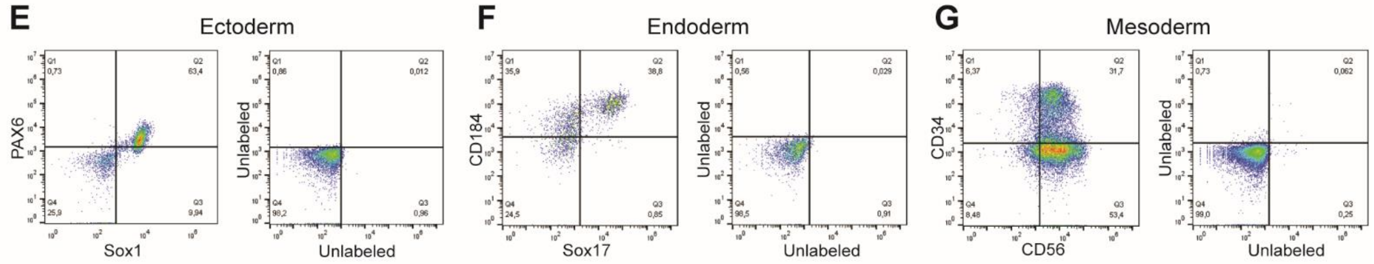

Supplementary Fig. 1 Quality control data for iPSC line from *VPS35* patient 1. (**A**) G-banding analysis showed a normal karyotype. (**B**) iPSCs on day 1 and 2 after thawing showed typical iPSC morphology as visualized by brightfield microscopy. (**C**) Short tandem repeat (STR) analysis on DNA extracted from *VPS35* patient 1 skin fibroblasts and corresponding undifferentiated iPSCs confirmed genomic identity. (**D**) Flow cytometry analysis showed expression of pluripotency markers Oct4, Sox2, Tra-1-81 and SSEA4 and low expression of differentiation marker SSEA1. Cells were gated against unstained cells. (**E-G**) Flow cytometry analysis showed expression of ectodermal markers Sox1 and PAX6 (**E**), endodermal markers CD184 and Sox17 (**F**) and mesodermal markers CD134 and CD56 (**G**).

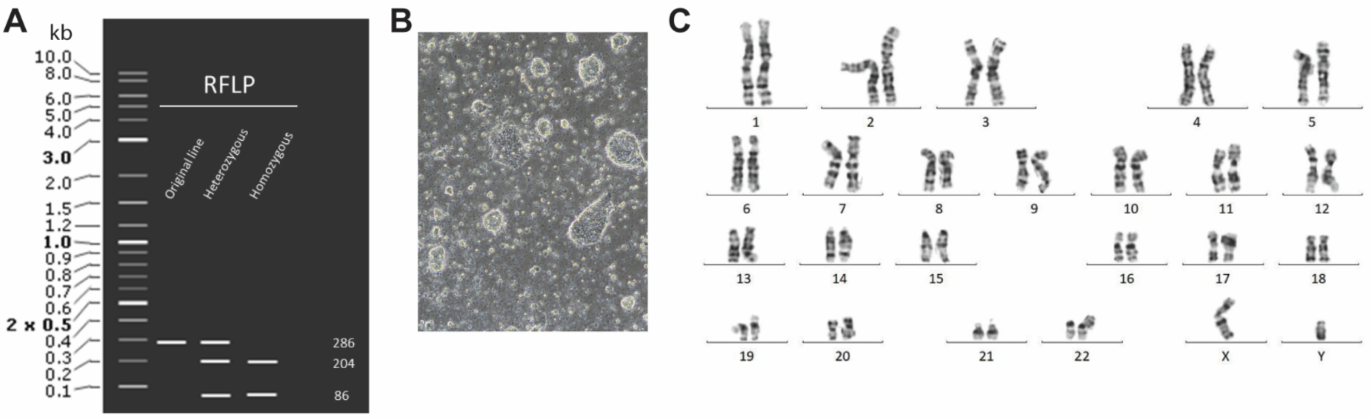

D

	VPS35 mutant fibroblasts		Isogenic control iPSCs	
Marker	Allele 1	Allele 2	Allele 1	Allele 2
AMEL	X	Y	X	Y
CSF1PO	11	12	11	12
D13S317	12		12	
D16S539	11	12	11	12
D18S51	12	16	12	16
D19S433	14		14	
D21S11	30	31.2	30	31.2
D2S1338	17	24	17	24
D3S1358	15	18	15	18
D5S818	11	12	11	12
0D7S820	10	11	10	11
D8S1179	13	16	13	16
FGA	23	25	23	25
TH01	6	9.3	6	9.3
TPOX	8		8	
vWA	18		18	

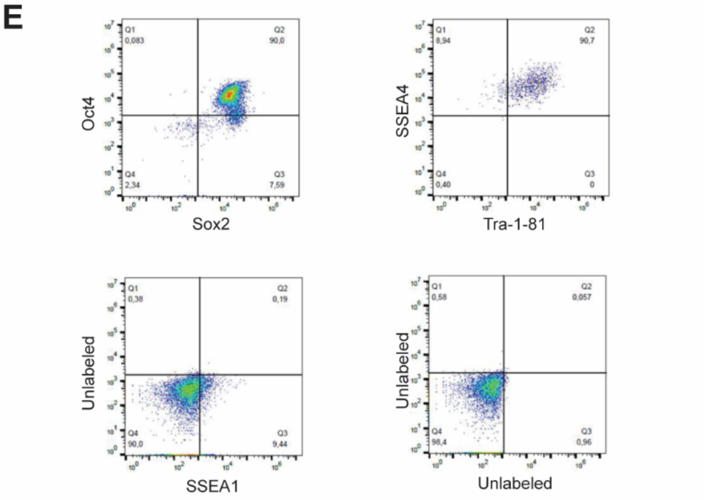

Supplementary Fig. 2 Quality control data for isogenic control iPSCs for *VPS35* Patient 1. (**A**) Restriction fragment length polymorphism (RFLP) analysis detected a homozygous gene-corrected clone (further confirmed by sanger sequencing). (**B**) iPSCs on day 1 after thawing showed typical iPSC morphology as visualized by brightfield microscopy. (**C**) G-banding analysis showed a normal karyotype. (**C**) Short tandem repeat (STR) analysis on DNA extracted from *VPS35* patient 1 skin fibroblasts and undifferentiated isogenic control iPSCs confirmed genomic identity. (**D**) Flow cytometry analysis showed expression of pluripotency markers Oct4, Sox2, Tra-1-81 and SSEA4 and low expression of differentiation marker SSEA1. Cells were gated against unstained cells.

A

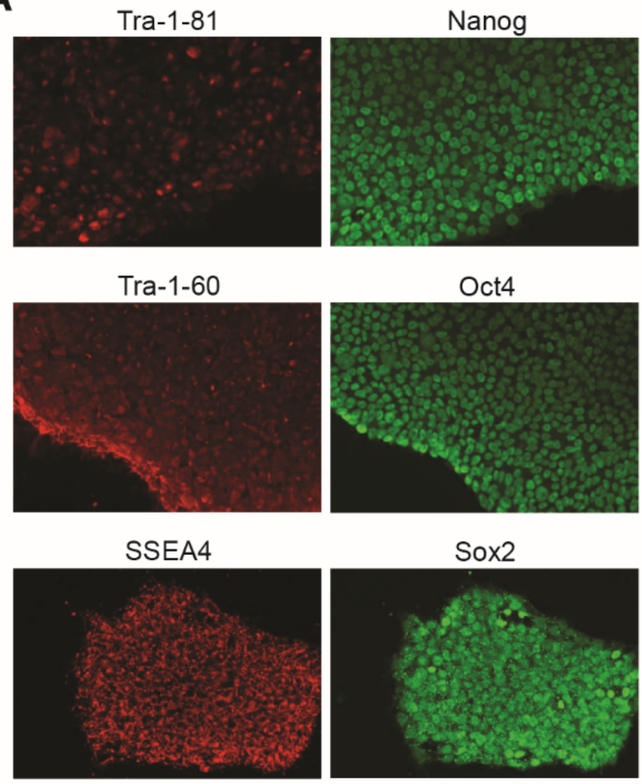

B

	Scorecard Δ Score
Self-renewal	-2.75
Ectoderm	2.08
Mesoderm	1.08
Endoderm	1.45

C

	Ctrl4 fibroblasts	Ctrl4 iPSCs
	Allele1/allele2	Allele1/allele2
C___1563023_10	VIC/VIC	VIC/VIC
C___1801627_20	FAM/FAM	FAM/FAM
C___2728408_10	VIC/VIC	VIC/VIC
C___1250735_20	FAM/FAM	FAM/FAM
C__15935210_10	FAM/FAM	FAM/FAM
C___7431888_10	VIC/VIC	VIC/VIC
C___3227711_10	VIC/FAM	VIC/FAM
C___1902433_10	VIC/VIC	VIC/VIC
C__30044763_10	VIC/FAM	VIC/FAM
C__31386842_10	VIC/FAM	VIC/FAM
C__33211212_10	VIC/FAM	VIC/FAM
C__26524789_10	VIC/VIC	VIC/VIC
C__11821218_10	VIC/FAM	VIC/FAM
C_____43852_10	VIC/FAM	VIC/FAM
C___1670459_10	VIC/VIC	VIC/VIC
C___8924366_10	FAM/FAM	FAM/FAM
C___1007630_10	VIC/VIC	VIC/VIC
C__11522992_10	FAM/FAM	FAM/FAM
C___7421900_10	VIC/VIC	VIC/VIC
C__10076371_10	FAM/FAM	FAM/FAM
C__26546714_10	VIC/FAM	VIC/FAM
C___1122315_10	VIC/FAM	VIC/FAM
C__27402849_10	VIC/VIC	VIC/VIC
C___7457509_10	VIC/FAM	VIC/FAM
C__29619553_10	FAM/FAM	FAM/FAM
C__11710129_10	VIC/FAM	VIC/FAM
C___2953330_10	VIC/FAM	VIC/FAM
C___1027548_20	UND	UND
C___8850710_10	VIC/VIC	VIC/VIC
C___1083232_10	NOAMP	NOAMP
C__16205730_10	VIC/VIC	VIC/VIC
C___8938211_20	NOAMP	NOAMP

Supplementary Fig. 3 Quality control data for Ctrl4 iPSC line. (**A**) Immunofluorescence of colonies showed expression of the pluripotency markers Tra-1-81, Nanog, Tra-1-60, Oct4, SSEA4 and Sox2. (**B**) ScoreCard results of trilineage differentiation potential confirmed the capacity to differentiate towards all three germ layers. (**C**) Analysis of genomic identity of the Ctrl4 skin fibroblasts and corresponding undifferentiated iPSCs using single nucleotide polymorphism (SNP) analysis and TaqMan Genotyper software.

Supplementary Table 1. Clinical characteristics of *VPS35* patient 1

	***VPS35* patient 1**	**Healthy controls**
**H&Y stage (off medication)**	2	NA
**MDS-UPDRS I**	7	3.1 ± 2.4
**MDS-UPDRS II**	12	0.5 ± 1.3
**MDS-UPDRS III (off medication)**	16	3.1 ± 2.1
**MDS-UPDRS IV**	0	NA
**LED (mg)**	400	NA
**MoCA**	27	28.6 ± 1.1
**AVLT ratio**	1	0.89 ± 0.09
**SDMT**	31	50.6 ± 8.2
**TMT-A**	22	22.5 ± 7.6
**TMT-B**	73	54.3 ± 19.8
**Benton H**	25	26.1 ± 2.9
**GDS**	0	1.2 ± 1.8
**PAS**	1	3.6 ± 3.9
**SCOPA-Sleep**	7	5.5 ± 4.4
**RBD1Q (yes/no)**	No	0/20
**SCOPA-AUT**	11	6.9 ± 3.7
**QuIP**	11	7.0 ± 7.1
**UPSIT**	26	31.2 ± 3.5

Clinical scores of *VPS35* patient 1 were obtained at the age of 61 years. For comparison, scores in 20 age-matched healthy controls (59.6 ± 8.2 years; 6 females, 14 males) are listed, as previously collected in-house as part of a clinical study.[27] Control data are given as mean ± SD. H&Y, Hoehn and Yahr stage; MDS-UPDRS, Movement Disorder Society–Unified Parkinson's Disease Rating Scale; LED, total daily levodopa equivalent dose; MoCA, Montreal Cognitive Assessment; AVLT, Auditory Verbal Learning Test, score A7/A5; SDMT, Symbol Digit Modalities Test; TMT-A, Trail Making Test Part A; TMT-B, Trail Making Test Part B; Benton H, Benton Judgment of Line Orientation test Part H; GDS, Geriatric Depression Scale; PAS, Parkinson's Anxiety Scale; SCOPA-Sleep, Scales for Outcomes in Parkinson's Disease–Sleep; SCOPA-AUT, Scales for Outcomes in Parkinson's Disease–Autonomic; RBD1Q, REM Sleep Behavior Disorder Single-Question Screen; QuIP, Questionnaire for Impulsive Disorders Questionnaire; UPSIT, University of Pennsylvania Smell Identification Test; NA, not applicable.
