# Supplementary material for: VPS35 mutation inhibits PINK1/parkin-mediated mitophagy via increased LRRK2 kinase activity
Source: Brain. 2025 Oct 30;149(7):2363–79. doi: 10.1093/brain/awaf414 (PMC13337223; doi:10.1093/brain/awaf414)
Supplement: awaf414_Supplementary_Data [file awaf414_supplementary_data.zip › brain-2025-01360-File011.pdf]

UNEDITED BLOTS

Red boxes indicate cropped areas shown in figures.

Unedited blots for Fig. 2C.

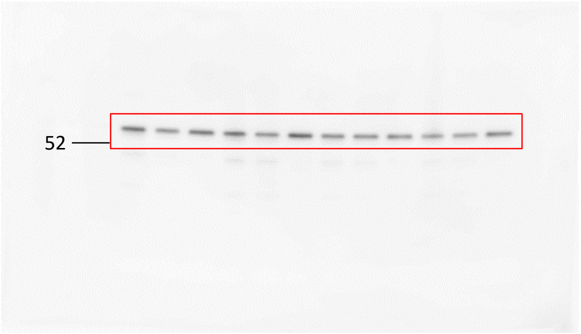

HSP60

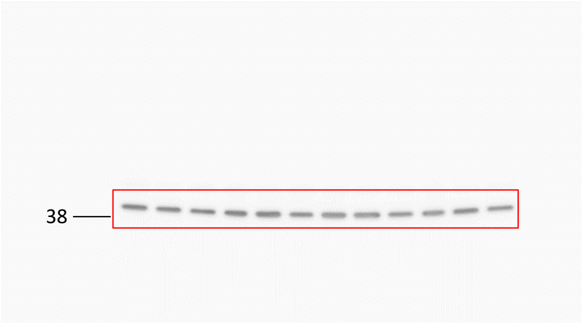

β-actin

Unedited blots for Fig. 4A.

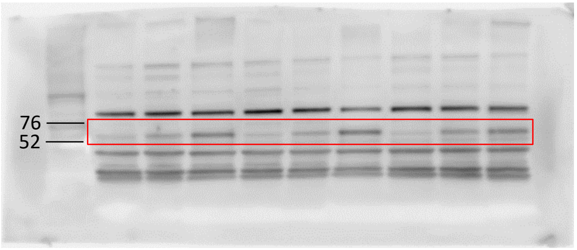

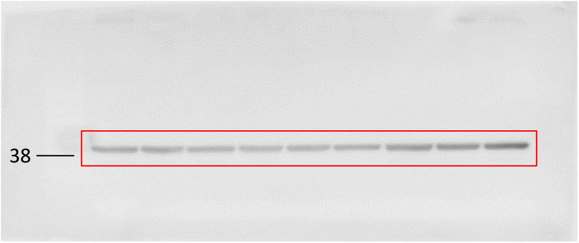

Unedited blots for Fig. 4C.

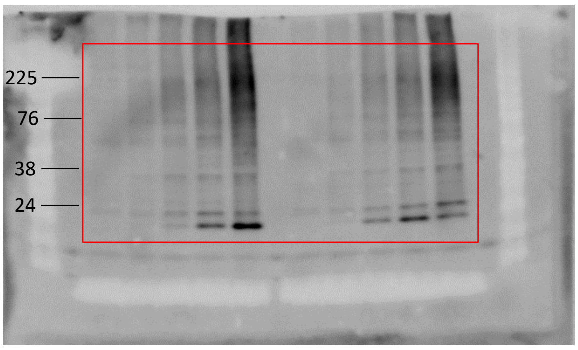

p-Ub

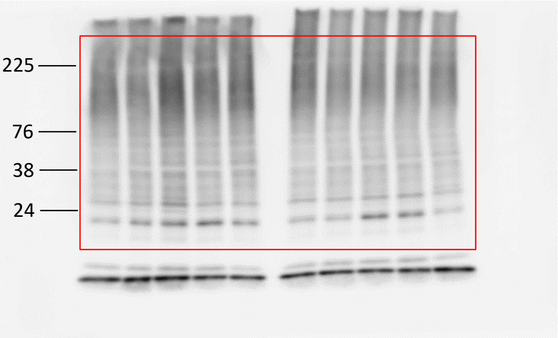

Ub

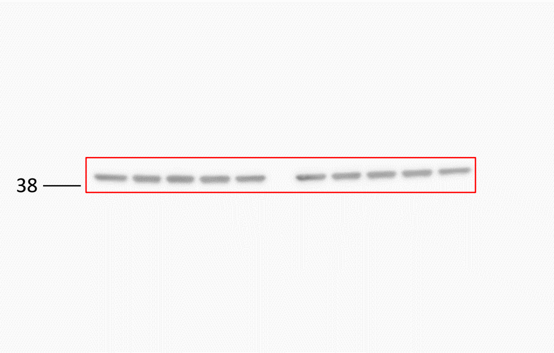

β-actin

Unedited blots for Fig. 4E: Blot Ctrl1 – Ctrl 2 – *PRKN* – *VPS35* Pt1. The membrane was cut in two prior to incubation with antibody; the upper part was incubated with MFN2 antibody and the lower part with β-actin antibody.

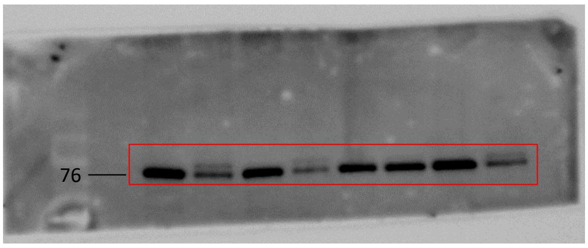

MFN2 (*Dark exposure*)

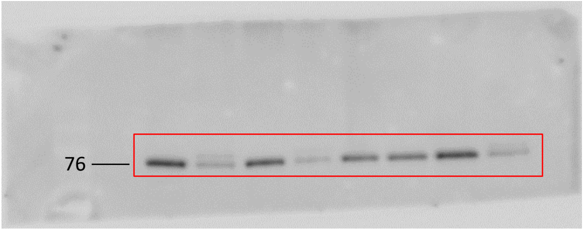

MFN2 (*Light exposure*)

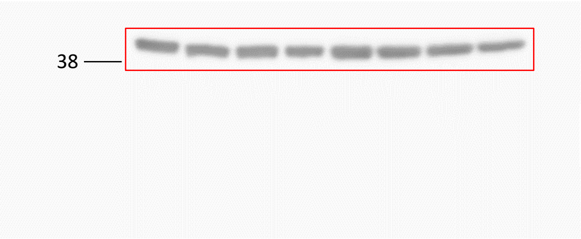

β-actin

Unedited blots for Fig. 4E: Blot Ctrl3 – *VPS35* Pt2. The membrane was cut in two prior to incubation with antibody; the upper part was incubated with MFN2 antibody and the lower part with β-actin antibody.

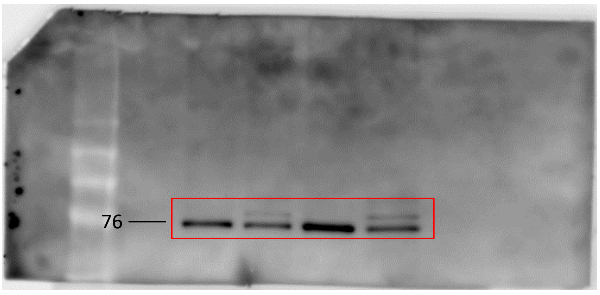

MFN2 (*Dark exposure*)

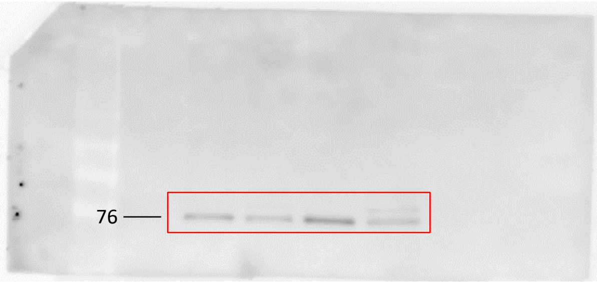

MFN2 (*Light exposure*)

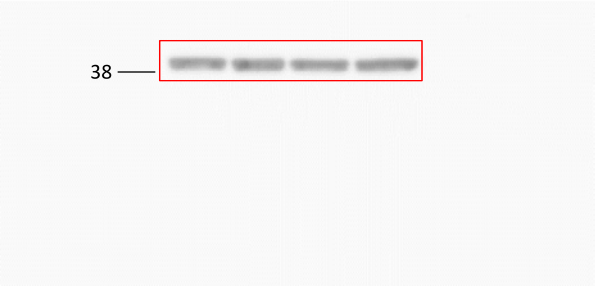

β-actin

Unedited blots for Fig. 5A.

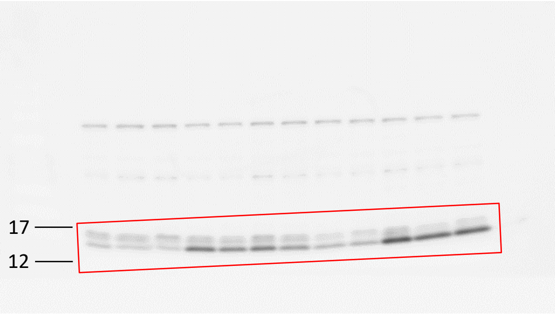

LC3

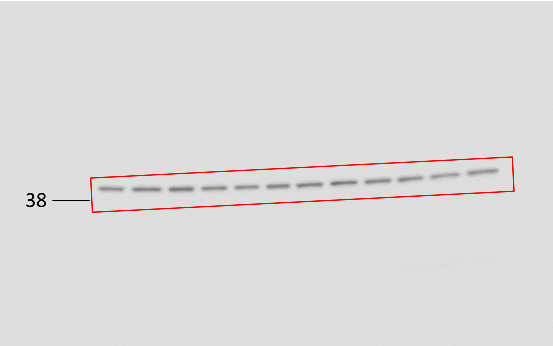

β-actin

Unedited blots for Fig. 6A.

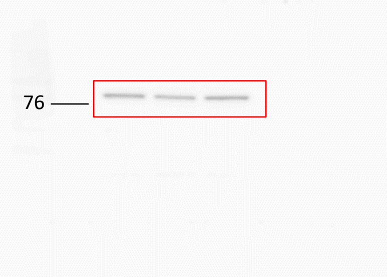

VPS35

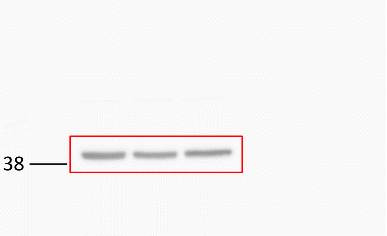

β-actin

Unedited blots for Fig. 6C: siRNA1.

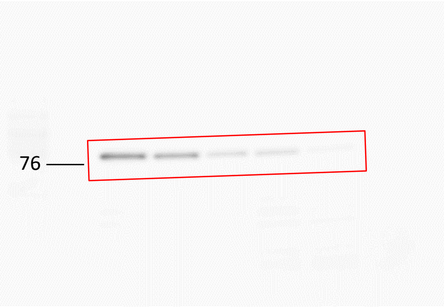

VPS35

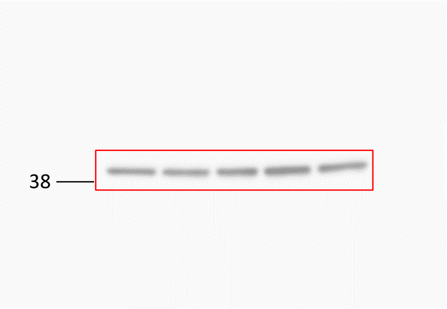

β-actin

Unedited blots for Fig. 6C: siRNA2.

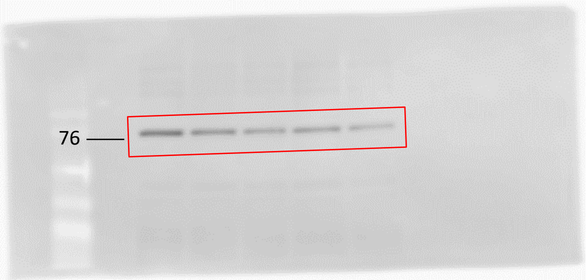

VPS35

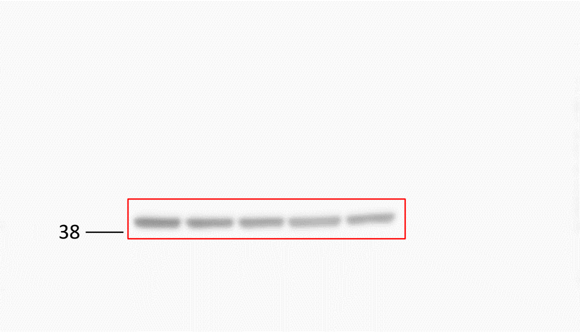

β-actin

Unedited blots for Fig. 6G.

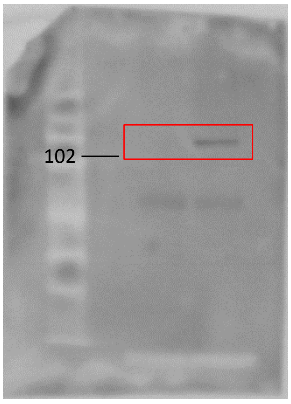

tGFP

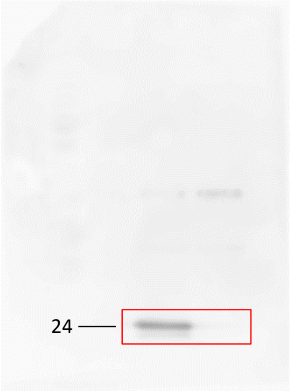

mGFP

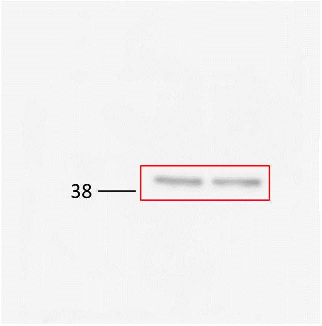

β-actin

Unedited blots for Fig. 6J: IsoCtrl – *VPS35* Pt1.

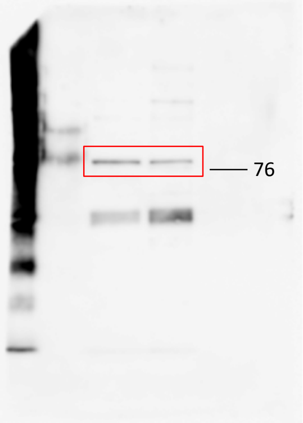

VPS35

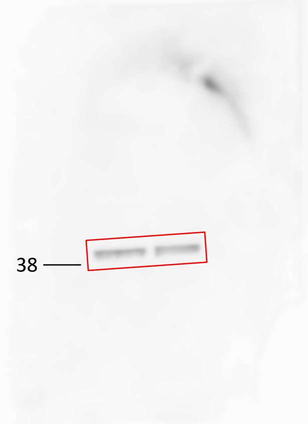

β-actin

Unedited blots for Fig. 6J: Ctrl4 – *VPS35* Pt2.

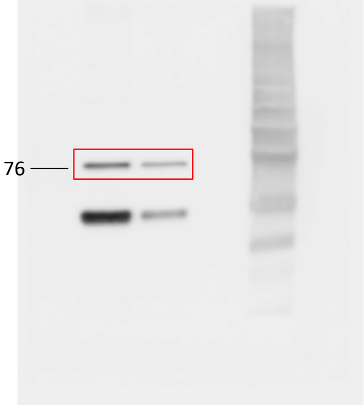

VPS35

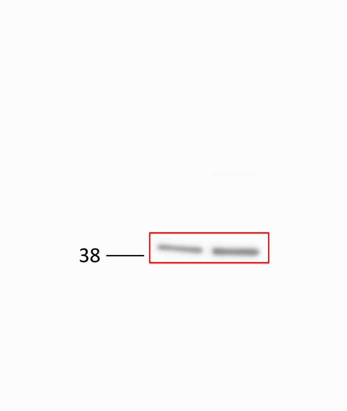

β-actin

Unedited blots for Fig. 6L.

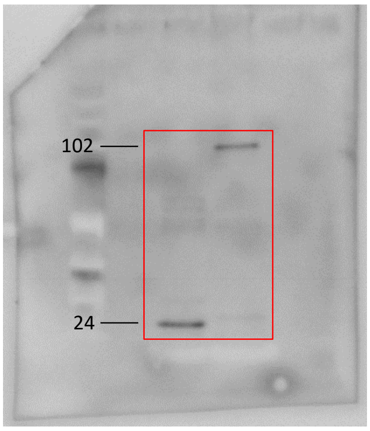

mGFP

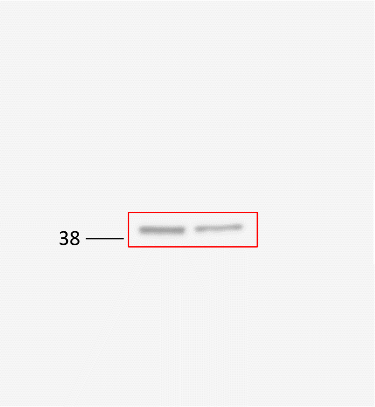

β-actin

Unedited blots for Fig. 7E.

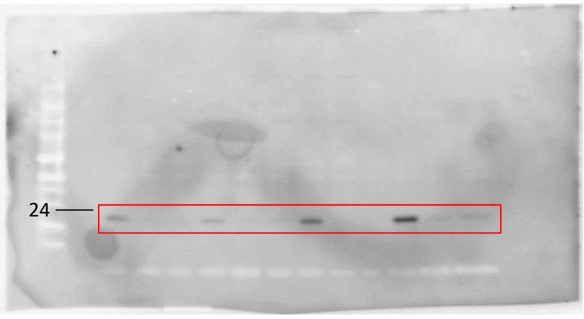

p-RAB10

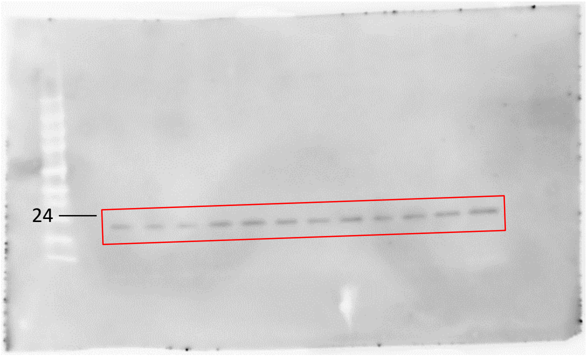

RAB10

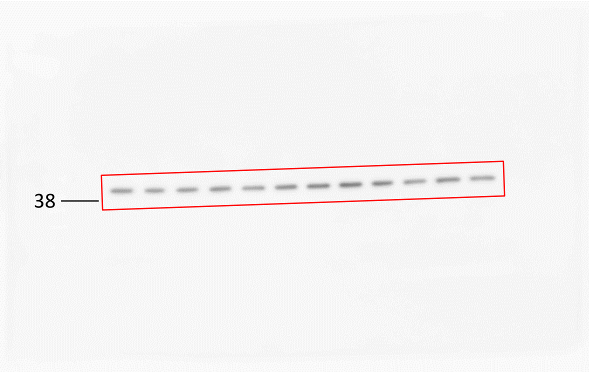

β-actin

Unedited blots for Fig. 7G. The membrane was cut in two prior to incubation with antibody; the upper part was incubated with VPS35 antibody and the lower part with p-RAB10 or RAB10 and β-actin antibody.

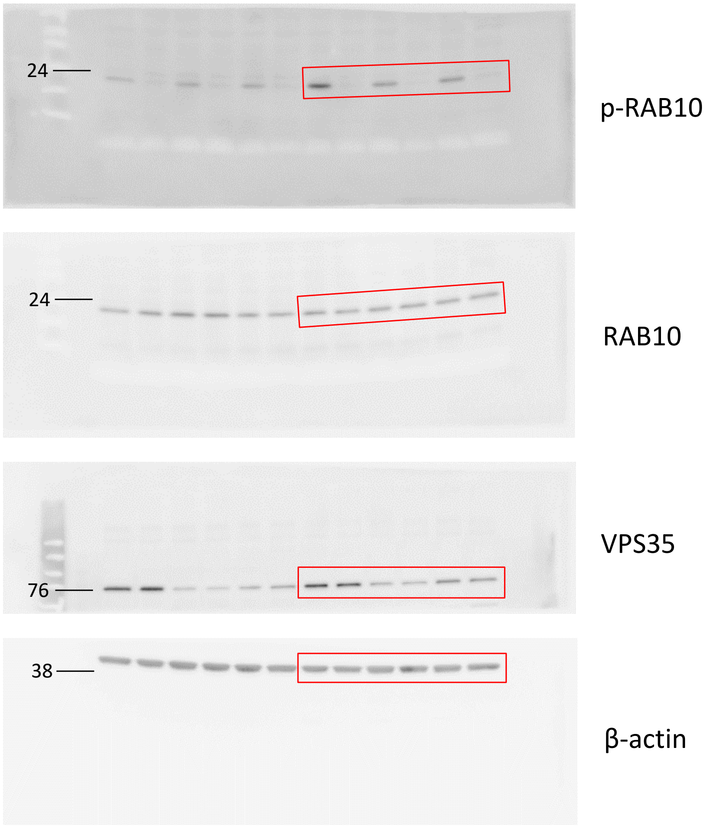

Unedited blots for Fig. 7I.

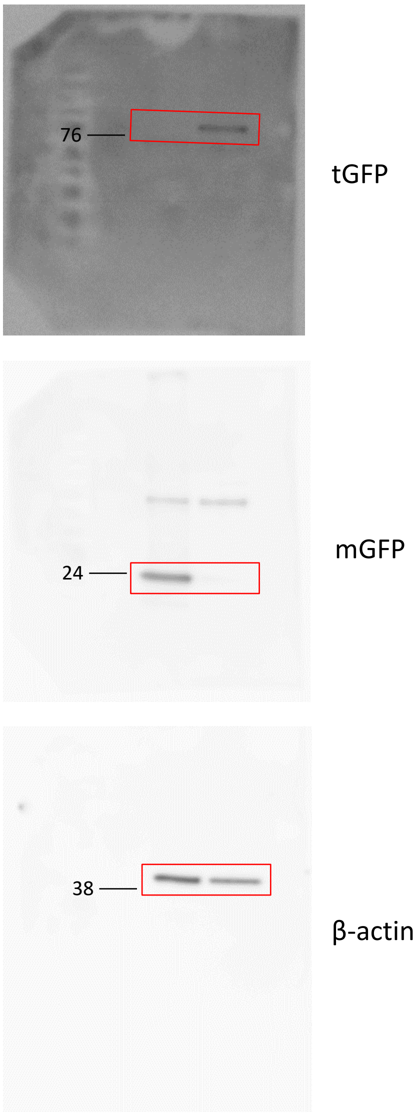

Unedited blots for Fig. 7L.

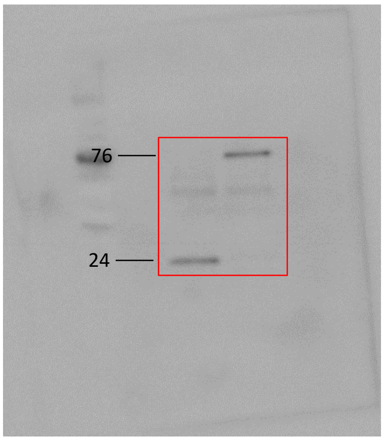

mGFP

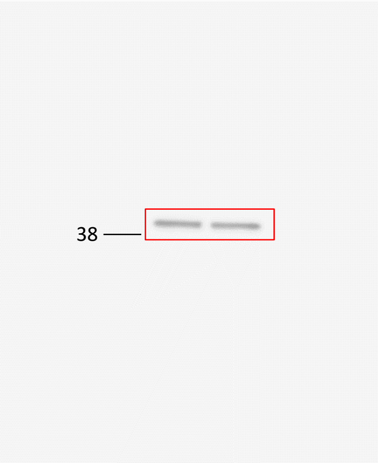

β-actin
